# The dynamics of evolutionary rescue from a novel pathogen threat in a host metapopulation

**DOI:** 10.1038/s41598-021-90407-z

**Published:** 2021-05-25

**Authors:** Jing Jiao, Nina Fefferman

**Affiliations:** 1 National Institute for Mathematical and Biological Synthesis, The University of Tennessee, 1122 Volunteer Blvd., Suite 106, Knoxville, TN 37996 USA; 2 Department of Biological Science, Florida State University, 319 Stadium Dr, Tallahassee, FL 32304 USA; 3 Ecology & Evolutionary Biology, The University of Tennessee, 1416 Circle Drive, Knoxville, TN 37996 USA

**Keywords:** Ecology, Evolution, Diseases

## Abstract

When a novel disease strikes a naïve host population, there is evidence that the most immediate response can involve host evolution while the pathogen remains relatively unchanged. When hosts also live in metapopulations, there may be critical differences in the dynamics that emerge from the synergy among evolutionary, ecological, and epidemiological factors. Here we used a Susceptible-Infected-Recovery model to explore how spatial and temporal ecological factors may drive the epidemiological and rapid-evolutionary dynamics of host metapopulations. For simplicity, we assumed two host genotypes: wild type, which has a positive intrinsic growth rate in the absence of disease, and robust type, which is less likely to catch the infection given exposure but has a lower intrinsic growth rate in the absence of infection. We found that the robust-type host would be strongly selected for in the presence of disease when transmission differences between the two types is large. The growth rate of the wild type had dual but opposite effects on host composition: a smaller increase in wild-type growth increased wild-type competition and lead to periodical disease outbreaks over the first generations after pathogen introduction, while larger growth increased disease by providing more susceptibles, which increased robust host density but decreased periodical outbreaks. Increased migration had a similar impact as the increased differential susceptibility, both of which led to an increase in robust hosts and a decrease in periodical outbreaks. Our study provided a comprehensive understanding of the combined effects among migration, disease epidemiology, and host demography on host evolution with an unchanging pathogen. The findings have important implications for wildlife conservation and zoonotic disease control.

## Introduction

The field of conservation biology explicitly considers the likely impact that different threats or supportive management actions are likely to have on the persistence of populations. The evaluation of these factors can take an ecological perspective, considering demographic effects (e.g., population viability analysis^1,2^), or behavioral factors (e.g., Allee effects^3,4^), or can take an evolutionary perspective, considering effective population size^5–7^, genetic rescue^8,9^, or even evolutionary rescue^1,10^. One critical advance in our understanding of threatened populations came from considering species that live in potentially connected patches of habitat through dispersal. This shift from a focus on an isolated population to metapopulation dynamics, and the difference in survival across a landscape by ongoing extinction-recolonization processes, fundamentally altered our understanding of both ecological and evolutionary bounds on persistence^11–13^. One of the strengths of the focus on metapopulations in conservation biology is that it enabled the same framework to address a large variety of potential threats. Climate change, habitat loss, habitat degradation, over-exploitation of a harvested population, or even invasions of competitors or predators that all fit within the same framework, are accessible for study using the same theory, and can be analyzed using the same conceptual and computational tools^14–19^. There is, however, one type of threat that is not easily considered in the same way, even though metapopulation dynamics may prove equally critical in evaluating an endangered population’s likelihood of persistence: infectious pathogens.

Infectious pathogens have the potential to drive the rapid extinction of entire populations^20–22^. Further, increased human transportation, land use changes, and global climate change have led to more frequent cases of the introduction or establishment of pathogens into new host species globally^23–25^. It is becoming clear that conservation biology must explicitly consider the potential for disease to impact species persistence^26,27^. Infectious disease risks depend directly on shifts in the host population itself. Outbreak dynamics of an infection are determined by the effective reproductive number for a disease, which characterizes its ability to continue spreading. This reproductive number depends directly on the available size of the susceptible population of hosts^28–30^. This means that, in some cases, actions taking in support of a population may inadvertently boost the duration of an active outbreak. Within a metapopulation framework, conservation actions taken within a single patch may perversely permit transmission to other patches, whereas allowing local extinction may protect the remainder of the host metapopulation from disease exposure^20,31^. Of course, ongoing metapopulation dynamics can complicate outcomes themselves, even in the absence of human intervention, since between patch migration that acts to replenish the host population in a depopulated patch may also function as a mechanism for (re)introduction of disease^32–34^.

In the worst potential case for an endangered host population, in which the disease causes substantial mortality, there are only three potential epidemiological outcomes for a host population when neither the host nor pathogen evolve: (1) The host population could die out. (2) The host population is sufficiently decimated (or protected by sufficient herd immunity) to curtail disease transmission, and the disease dies out. In this case, the population may retain sufficient size or metapopulation dynamics to recover, so long as the disease is not reintroduced into the metapopulation from an outside source. (3) The population is sufficiently large to withstand the die-off associated with the initial outbreak of infection and long-term demographic and migration rates of the population are sufficient to both support and withstand constant, relatively low levels of disease transmission over time (i.e., the disease becomes endemic).

These three possible epidemiological outcomes all function within the context of host/pathogen ecology. However, that does not restrict the potential for evolutionary dynamics to co-occur. For most host–pathogen systems, the most likely observable evolutionary changes will occur in the pathogen^35–37^ (although host resistance to disease can also evolve^38,39^). This is due not only to the shorter generation time, but also to the highly variable nature of microbial reproduction (e.g., horizontal gene transfer^38,39^; high rates of antigenic mutation^40–42^ etc.). Theory further predicts that selective pressures due to epidemiological dynamics should favor strains with decreased pathogenicity over time^43–46^, meaning that host prospects for surviving under endemic threat, after initial epidemic die-offs may be better than if the pathogen remained unchanged over time. However, for the same reasons as the fluctuating risk of infection itself, the intensity of the selective pressure on the pathogen should wax and wane with host population sizes and patch dynamics.

In addition to the possibility of pathogen evolution and host–pathogen coevolution, there is also a potentially equally important possibility of host evolution, especially when a novel disease invades host population. Due to the accrual of mutations, a host population is likely to have a diversity of existing genotypes, some of which that are more resistant/ tolerant to disease than other types (e.g., some bat species are more tolerant to White Nose Syndrome than others^1^). Hence, in the sudden presence of the novel selective pressure of a disease, the host population has been observed to evolve while the pathogen remains relatively genetically stable (e.g., White Nose Syndrome and ranavirus^1,47^ are two of the best-known examples) over a conservation-relevant timeframe^10^. In fact, it may be no coincidence that these two well-known examples are of rapidly expanding invasive infections—entering into a novel host population, with an abundance of readily accessible susceptible individuals to infect. In these cases, there should be very little selective pressure on the pathogen^10^. This is because the host can be expected to have no co-evolved traits to combat the threat of the specific pathogen, meaning that it is unlikely for there to be a single, unified selective pressure from host defenses that skew the success of one pathogen strain over another. The emergence of random mutations should remain the dominant route of genotypic shifting among the pathogen early after introduction into a novel host population; the pathogenic invasion behaves equivalently to prey under a scenario of predator release. The hosts, on the other hand, in this case may face a strong immediate selective pressure and may therefore be expected to evolve on a more rapid timescale then their invading pathogen if they are to survive. However, both empirical investigations and theoretical work across different systems to look for evidence of such rapid evolutionary rescue effects (e.g., White Nose Syndrome^1^) are still rare.

These unique features of infectious disease threats move both the ecological and evolutionary theories for population persistence of metapopulations outside of the realms that can be well-studied by existing tools. When trying to understand how endangered populations might respond to the threat of infection, it is no longer sufficient to extrapolate from models that consider the ecology, evolution, or epidemiology of the system in isolation. For example, host migration under metapopulation structure can (re)introduce disease to local patch, which could lead to evolutionary rescue and modify disease epidemics therein. Hence, we require a synergistic model that can incorporate metapopulation dynamics, shifting selective pressures, and disease transmission simultaneously.

We here present a classic Susceptible-Infected-Recovered epidemiological model in a host metapopulation structure, in which disease cannot exist outside of the target host, therefore only the host can migrate among patches (potentially carrying infection with them; see a similar model structure in previous study^10^). We specifically explored how each of these three critical, inextricable processes (i.e., metapopulation dynamics, shifting selective pressures, and disease transmission) may shape the persistence of species as novel pathogens arise and existing pathogens spread into novel geographic regions and affect new host populations. For simplicity, we first considered a “stepping-stone” host metapopulation model with clockwise disease migration among patches, in which the timing and direction of disease spreading can be well controlled. Some human migration, or ocean transport systems may follow this simple structure^48,49^. To test the generality of the model results, we then also gradually added edges to the “stepping-stone” structure and studied the sensitivity of the model dynamics towards more complicated host metapopulation structures.

## Model

1 $$\frac{{dS_{ji} }}{dt} = B_{ji} - \sum \limits_{{h \in \left[ {W,R} \right]}} \beta_{hi} I_{jh} S_{ji} - \mu_{i} S_{ji} + m_{ji}^{S}$$

2 $$\frac{{dI_{ji} }}{dt} = \sum \limits_{{h \in \left[ {W,R} \right]}} \beta_{hi} I_{jh} S_{ji} - \left( {\alpha_{i} + \mu_{i} + \gamma_{i} } \right)I_{ji} + m_{ji}^{I}$$

3 $$\frac{{dR_{ji} }}{dt} = \gamma_{i} I_{ji} - \mu_{i} R_{ji} + m_{ji}^{R}$$

where the state variables, $S_{ji}$, $I_{ji}$ and $R_{ji}$ are the numbers of susceptible, infected and recovered hosts of genotype *i* at patch *j*, respectively. *i* is either Wild type (W) or robust type (R). $\beta_{hi}$ describes the transmission rate from Infected genotype *h* to *i*, $\mu_{i}$, $\alpha_{i}$ and $\gamma_{i}$ are the natural mortality, disease-induced mortality and recovery rate in genotype *i*, respectively. Recovered hosts are assumed to remain immune to the disease for the remainder of their lifetime, but their offspring do not inherit this adaptive immunity (i.e., no vertical transmission). $B_{ji}$ represents all newborns that have genotype *i* and are from randomly mated in host population at patch *j.* When mating happens within same-type host pairs, all the newborns are assumed to be susceptibles of the same type as their parents. When mating happens across types, we assume that 50% offspring are robust type and 50% are wild type, such that

4 $$B_{ji} = \sum \limits_{{h \in \left[ {W,R} \right]}} L_{{S_{ji} S_{jh} }} + \sum \limits_{{h \in \left[ {W,R} \right]}} L_{{S_{ji} I_{jh} }} + \sum \limits_{i \ne h} L_{{S_{jh} I_{ji} }} + \sum \limits_{{h \in \left[ {W,R} \right]}} L_{{S_{ji} R_{jh} }} + \sum \limits_{i \ne h} L_{{S_{jh} R_{ji} }} + \sum \limits_{{h \in \left[ {W,R} \right]}} L_{{I_{ji} R_{jh} }} + \sum \limits_{i \ne h} L_{{I_{jh} R_{ji} }} + \sum \limits_{{h \in \left[ {W,R} \right]}} L_{{I_{ji} I_{jh} }} + \sum \limits_{{h \in \left[ {W,R} \right]}} L_{{R_{ji} R_{jh} }}$$

in which $L$ describes the newborns from each pair of mating combinations (within and between susceptible and infected for both genotypes: e.g.,${ }L_{{S_{ji} S_{jh} }}$ is the newborn number from the mating between $S_{ji}$ and $S_{jh}$). The detailed equations are:

5 $$L_{{S_{ji} S_{ji} }} = \frac{{S_{ji}^{2} }}{{P_{j} }}r_{i} \left( {1 - \frac{{P_{j} }}{{K_{j} }}} \right)$$

6 $$L_{{S_{ji} S_{jh} }} = \frac{{S_{ji} S_{jh} }}{{P_{j} }}\left( {\frac{{r_{i} + r_{h} }}{2}} \right)\left( {1 - \frac{{P_{j} }}{{K_{j} }}} \right)\;\;\;{\text{where}}\;i \ne h$$

7 $$L_{{S_{ji} I_{ji} }} = \frac{{2S_{ji} I_{ji} }}{{P_{j} }}\left( {\frac{{r_{i} + r_{id} }}{2}} \right)\left( {1 - \frac{{P_{j} }}{{K_{j} }}} \right)$$

8 $$L_{{S_{ji} I_{jh} }} = \frac{{S_{ji} I_{jh} }}{{P_{j} }}\left( {\frac{{r_{i} + r_{hd} }}{2}} \right)\left( {1 - \frac{{P_{j} }}{{K_{j} }}} \right)\;\;\;{\text{where}}\;i \ne h$$

9 $$L_{{S_{ji} R_{ji} }} = \frac{{2S_{ji} R_{ji} }}{{P_{j} }}\left( {\frac{{r_{i} + r_{ir} }}{2}} \right)\left( {1 - \frac{{P_{j} }}{{K_{j} }}} \right)$$

10 $$L_{{S_{ji} R_{jh} }} = \frac{{S_{ji} R_{jh} }}{{P_{j} }}\left( {\frac{{r_{i} + r_{hr} }}{2}} \right)\left( {1 - \frac{{P_{j} }}{{K_{j} }}} \right)\;\;\;{\text{where}}\,i \ne h$$

11 $$L_{{I_{ji} I_{ji} }} = \frac{{I_{ji}^{2} }}{{P_{j} }}r_{id} \left( {1 - \frac{{P_{j} }}{{K_{j} }}} \right)$$

12 $$L_{{I_{ji} I_{jh} }} = \frac{{I_{ji} I_{jh} }}{{P_{j} }}\left( {\frac{{r_{id} + r_{hd} }}{2}} \right)\left( {1 - \frac{{P_{j} }}{{K_{j} }}} \right)\;\;\;{\text{where}}\;i \ne h$$

13 $$L_{{I_{ji} R_{ji} }} = \frac{{2I_{ji} R_{ji} }}{{P_{j} }}\left( {\frac{{r_{id} + r_{ir} }}{2}} \right)\left( {1 - \frac{{P_{j} }}{{K_{j} }}} \right)$$

14 $$L_{{I_{ji} R_{jh} }} = \frac{{I_{ji} R_{jh} }}{{P_{j} }}\left( {\frac{{r_{id} + r_{hr} }}{2}} \right)\left( {1 - \frac{{P_{j} }}{{K_{j} }}} \right)\;\;\;{\text{where}}\;i \ne h$$

15 $$L_{{R_{ji} R_{ji} }} = \frac{{R_{ji}^{2} }}{{P_{j} }}r_{ir} \left( {1 - \frac{{P_{j} }}{{K_{j} }}} \right)$$

16 $$L_{{R_{ji} R_{jh} }} = \frac{{R_{ji} R_{jh} }}{{P_{j} }}\left( {\frac{{r_{ir} + r_{hr} }}{2}} \right)\left( {1 - \frac{{P_{j} }}{{K_{j} }}} \right)\;{\text{where}}\;i \ne h$$

Here, $r_{i}$, $r_{id}$ and $r_{ir}$ are the growth rate of susceptible, infected and recovered hosts in genotype *i*, respectively. *h* and *i* are either Wild type (W) or Robust (R). $P_{j}$ is the total number of hosts (the sum of all susceptible and infected hosts) in patch *j*:

17 $$P_{j} \left( t \right) = \sum \limits_{{i \in \left[ {W,R} \right]}} S_{ji} \left( t \right) + \sum \limits_{{i \in \left[ {W,R} \right]}} I_{ji} \left( t \right) + \sum \limits_{{i \in \left[ {W,R} \right]}} R_{ji} \left( t \right)$$

$m_{ji}^{S}$, $m_{ji}^{I}$ and $m_{ji}^{R}$ describe the net immigration of genotype *i* to patch *j*:

18 $$m_{ji}^{S} = \sum \limits_{{k \in \left[ {1,N} \right]/\left\{ j \right\}}} \left[ {mig\left( {k,j} \right)S_{ki} - mig\left( {j,k} \right)S_{ji} } \right]$$

in which $mig\left( {k,j} \right)$ describes the migration from patch *k* to *j*. Replacing *S* by *I* or *R*, we can get $m_{ji}^{I}$ or $m_{ji}^{R}$. All parameter notations, description, units and values for simulation are listed in Table 1. The model was run for 100,000 steps, which equated to 50 host generations to consider a reasonable timeframe for conservation purposes. We assumed that the initial balance of host types was equal to keep simple disease dynamics in the absence of migration and leave it to future work to consider how the different skew of host type across different patches could impact the epidemiological and evolutionary metapopulation dynamics of the system.

**Table 1:** All parameter notation, description, unit in the model and values for simulation.

Parameters	Description	Unit	Value
*r*_*i*_	growth rate of the susceptible in host type *i*	1/time	*rW* = 0.2, *r*_*R*_ = 0.15
*r*_*id*_	growth rate of infected in host type *i*	1/time	0.01
*r*_*ir*_	growth rate of the recovered in host type *i*	1/time	0.2
α_*i*_	Disease-introduced mortality for host type *i*	1/time	0.05
μ_*i*_	natural mortality of host type *i*	1/time	0.01 or 0.05
β_ℎ*i*_	transmission from infected host type *i* to susceptible host type *h*	1/indiv./time	β_*RR*_ = β_*WR*_ = 0.000005, β_*RW*_ = β_*WW*_ = 0.0001
γ_*i*_	recover rate of host type i	1/time	0.05
*K*_*j*_	carrying capacity of host type *j*	indiv	3000

*i* = W or R, representing host genotype wild-type and robust type, respectively.

Through this model, we first studied the host dynamics in one isolated patch, which indicated the effects from disease epidemiology and host demography. We then extended our study to multiple patches and further explored how host dynamics also responded to between-patch migration when disease was initially introduced into only one patch. To easily control the direction of disease transmission among patches, we first assumed a clockwise transmission under a stepping-stone metapopulation structure with two ends wrapped around. We specifically tested how subpopulation sizes (either wild type or robust), total population size (wild type + robust) and periodic outbreaks (due to disease reintroduction from other patches) changed under the influences of three pairs of parameter combinations: Transmission ratio vs. growth ratio in the absence of disease ($r_{W} /r_{R}$), migration rate vs. growth ratio without disease and transmission ratio vs. migration rate. We also relaxed the model assumption by adding an additional edge to the “stepping-stone” structure and explored the corresponding change in disease dynamics.

## Results

### one-patch dynamics in the absence of migration

From Eqs. (1–2), we analytically solved the basic reproduction number ($R_{0}$) in one isolated patch *j* using the next generation matrix method^48^:

19 $$R_{0} = \frac{{\beta_{W} B_{jW|I = 0} + \beta_{R} B_{jR|I = 0} }}{{\mu \left( {\alpha + \mu + \gamma } \right)}}$$

where $B_{jW|I = 0}$ and $B_{jR|I = 0}$ are reproductive rate of each genotype (*W* or *R*) in the absence of disease.

**Figure 1:**
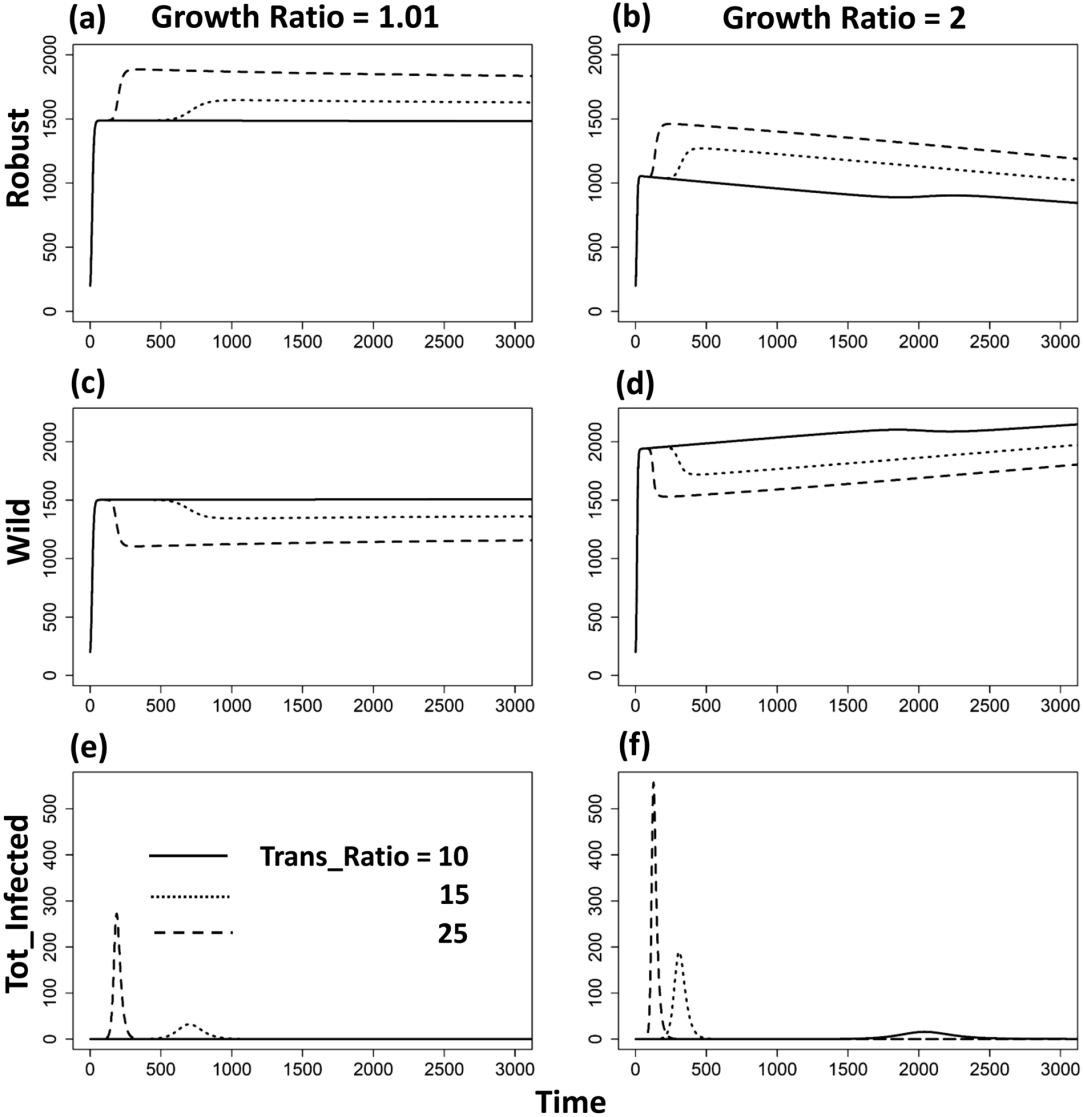
The dynamic changes of two host genotypes (Robust and Wild) and total infecteds under three levels of transmission ratio (10, 15 and 25, indicated by solid, dotted and dashed lines respectively) and two levels of growth ratio (1.01: left panels **a**,**c**,**e**; 2: right panels **b**,**d**,**f**) in one isolated patch. The robust type serves as the baseline transmission rate and growth rate of the susceptibles. All the other parameters are: *r*_*R*_ = 0.15, *r*_*Wd*_ = *r*_*Rd*_ = 0.01, *r*_*Wr*_ = *r*_*Rr*_ = 0.2, α_*W*_ = α_*R*_ = 0.05, μ_*W*_ = μ_*R*_ = 0.0005, γ_*W*_ = γ_*R*_ = 0.05, β_*RR*_ = β_*WR*_ = 0.000005. Initial susceptibles of both types are 200, initial infected of both types are 0.0005, and initial recovereds of both types are 0.

Over time, the disease exhibited standard outbreak dynamics, with a single peak in incidence that burned through the population and then died out under the parameter set (see the two bottom panels in Fig. 1). Due to the absence of between-patch migration, the disease in one patch did not reoccur after dying out since there was no reintroduction from other patches.

During the growth phase of the outbreak (mostly in the first 300 timesteps), the robust type was selected for while representation of the wild type decreased due to its higher numbers of deaths as a result of disease (see the sudden increase in Robust (Fig. 1a,b) but decrease in wild type (Fig. 1c,d) when disease increases (see the first 300 timesteps in Fig. 1e,f)). The larger transmission ratio ($\beta_{W} /\beta_{R}$ with $\beta_{R}$ fixed) led to larger increase in disease ($R_{0}$ in Eq. (19) increased due to the increase of $\beta_{W} /\beta_{R}$; compare the three lines in Fig. 1f), thus, the stronger selection on host genotypes leading to evolutionary rescue effects, i.e., robust density increased more, and wild type decreased more, as the transmission rate increased (compare the three lines in Fig. 1b,d). With the lower growth ratio and transmission ratio (e.g., growth ratio = 1.01 and transmission ratio = 10), the system showed herd immunity without strong disease selection (see the almost constant sizes of both genotypes along time in Fig. 1a,c,e).

As the number of infected individuals decreased (after 400 timesteps in Fig. 1e,f), disease-driven selection on host genotypes was also reduced: i.e., the robust type lost its advantage (see the decrease of robust after 400 timesteps in Fig. 1a,b). Once the disease died out, the wild type then gradually increased in density due to its higher growth rate in the absence of disease (see the increase trend in Fig. 1c,d). The larger the growth ratio ($r_{W} /r_{R}$ with $r_{R}$ fixed) was, the reproduction in Wild type ($B_{jW|I = 0}$) was larger and disease selection was stronger (larger $R_{0}$ in Eq. 19), which led to a stronger increase in Robust type and large decrease in Wild type (compare the trends of the two genotypes in Fig. 1a–d).

Although a large growth ratio (r) would benefit the wild type in general, it could also reinforce disease-driven selection by increasing the total density of infected individuals. For example, around timestep = 130, the growth ratio at 2 experienced increased disease relative to the growth ratio at 1.01 (compare the maximum values of total infected individuals between Fig. 1e,f). This is because the large growth rate provided more susceptibles (via reproduction) who then caught and propagated the infection. In this case, a higher growth rate would benefit the robust type due to increased disease-driven selection. Therefore, the growth ratio could act either as a selective force against the wild type or the robust type, depending on the magnitude and relative demographic rates of the different host types.

### Multiple-patch dynamics with migration

#### Stepping-stone metapopulation with clockwise migration

Under the stepping-stone structure for the host metapopulation, between-patch migration could reintroduce disease to the patch(es) where it had previously died out. Depending on the epidemiological characteristics and the timing of disease moving among all patches, disease could be reinforced (i.e., $R_{0}$ increased after the inclusion of $\rho$; the rate at which disease is reintroduced into patches with already high prevalence of infection; see Eq. 20 in Appendix 1), lost (i.e., $R_{0}$ < 1 as Eq. (19) even in the presence of migration; because disease in one patch died out before being carried to any neighboring patch) or cycling (i.e., $R_{0}$ $\approx 1$ after the inclusion of $\rho$ and the effective reproductive number *R* $\approx 1$ in Fig. S3 in Appendix 1; disease in one patch is always successfully (re)introduced to at least one neighboring patch where it establishes before dying out in the parent patch, leading to repeated outbreaks in each patch over time).

In the case in which migration led to the reinforcement of disease, the robust type would be selected for across all patches due to its lower numbers of deaths due to disease (see Fig. S1 in Appendix 1); when disease died out in one patch without being carried to the next (thus dying out across the entire system), the wild type would gradually take over the system due to its higher growth rate (see Fig. S2 in Appendix 1); when disease showed periodical outbreaks through patches, the robust and wild types would alternate their domination of the system (i.e., the wild type dominated after the disease died out in each patch, but since the robust type remained extant, when disease was reintroduced by migration, the robust type would again increase; see Fig. S3 in Appendix 1).

**Figure 2:**
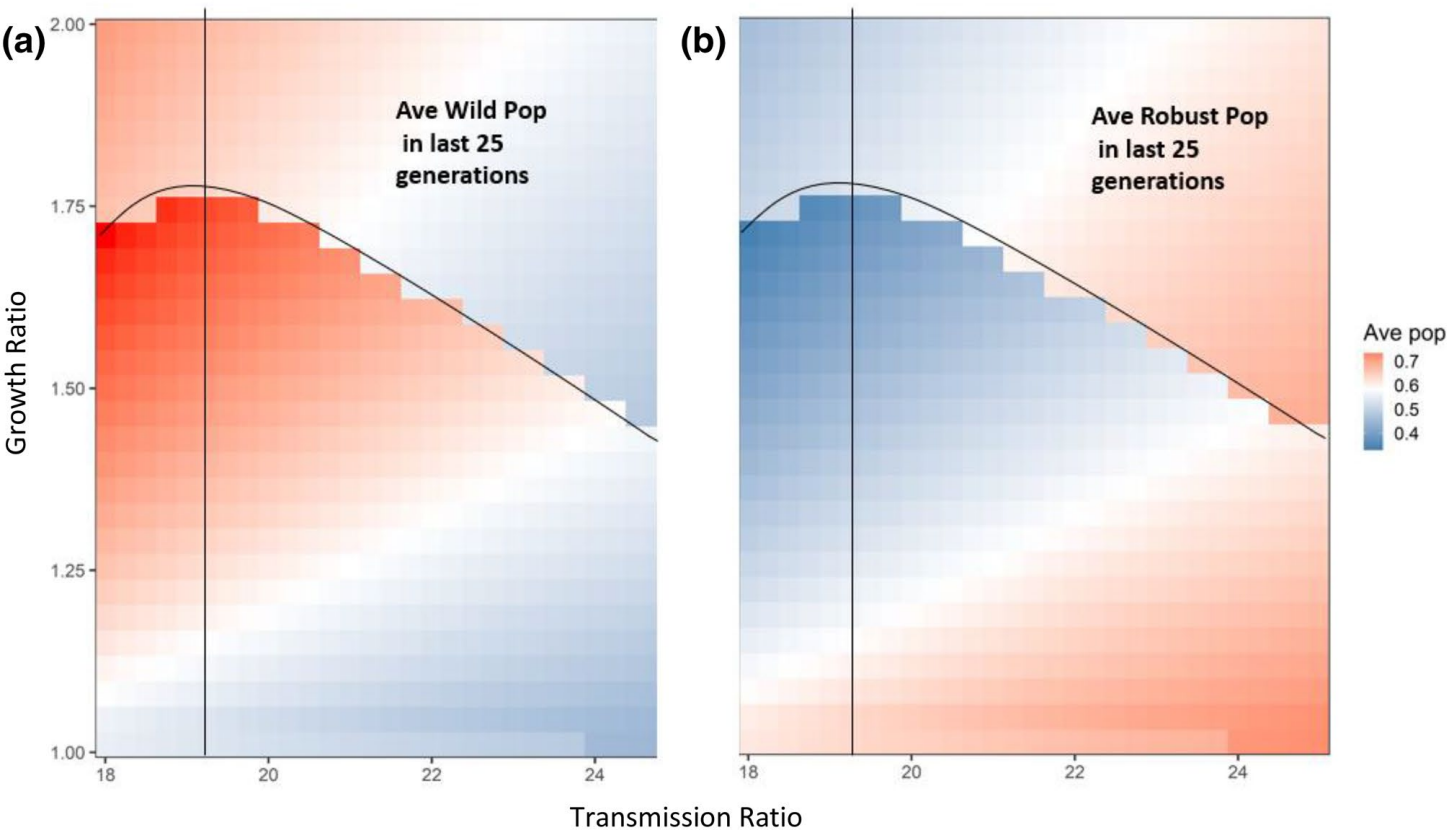
The standardized average population size (population size divided by carrying capacity) of both wild type (**a**) and robust type (**b**) in the last 25 generations (total simulated generations are 50) under the influences of transmission ratio and growth ratio at patch #30, where robust type provides the baseline growth rate and transmission rate. The color trend represents the average population size: from blue to white to red, average population sizes increase. All the other parameters are: *r*_*R*_ = 0.15, *r*_*Wd*_ = *r*_*Rd*_ = 0.01, *r*_*Wr*_ = *r*_*Rr*_ = 0.2, α_*W*_ = α_*R*_ = 0.05, μ_*W*_ = μ_*R*_ = 0.0005, γ_*W*_ = γ_*R*_ = 0.05, *β*_*RR*_ = *β*_*WR*_ = 0.000005 and *m*(*k*,*j*) = 0.000008. In focal patch #1, the initial susceptibles of both types are 200, infecteds are 5, and recovereds are 0. Other patches show similar patterns.

Over the last 25 generations, the growth ratio would first show an average increase but then an average decrease in the density of the wild type (see the color trend along y-axis in Fig. 2a and Fig. S4a in Appendix 2). The complementary pattern existed for the robust type (color trend along y-axis in Fig. 2b and Fig. S4b in Appendix 2). This is due to the dual functions of growth rate: increasing the competition from the wild type as well as strengthening disease-driven selective advantage of the robust type via producing more susceptible offspring for disease infection (similar to the pattern observed in the one patch scenario; compare the two genotypes and total infected individuals between Fig. 1a,b). As the growth ratio increased (see above the bifurcation line near growth ratio = 1.75 in Figs. 2 and Fig. S4 in Appendix 2), the increased prevalence of infection outweighed the increase in wild-type growth, showing selection favoring the robust type (compare the color trends in the areas along y-axis above the bifurcation line in Figs. 2 and Fig. S4 in Appendix 2).

**Figure 3:**
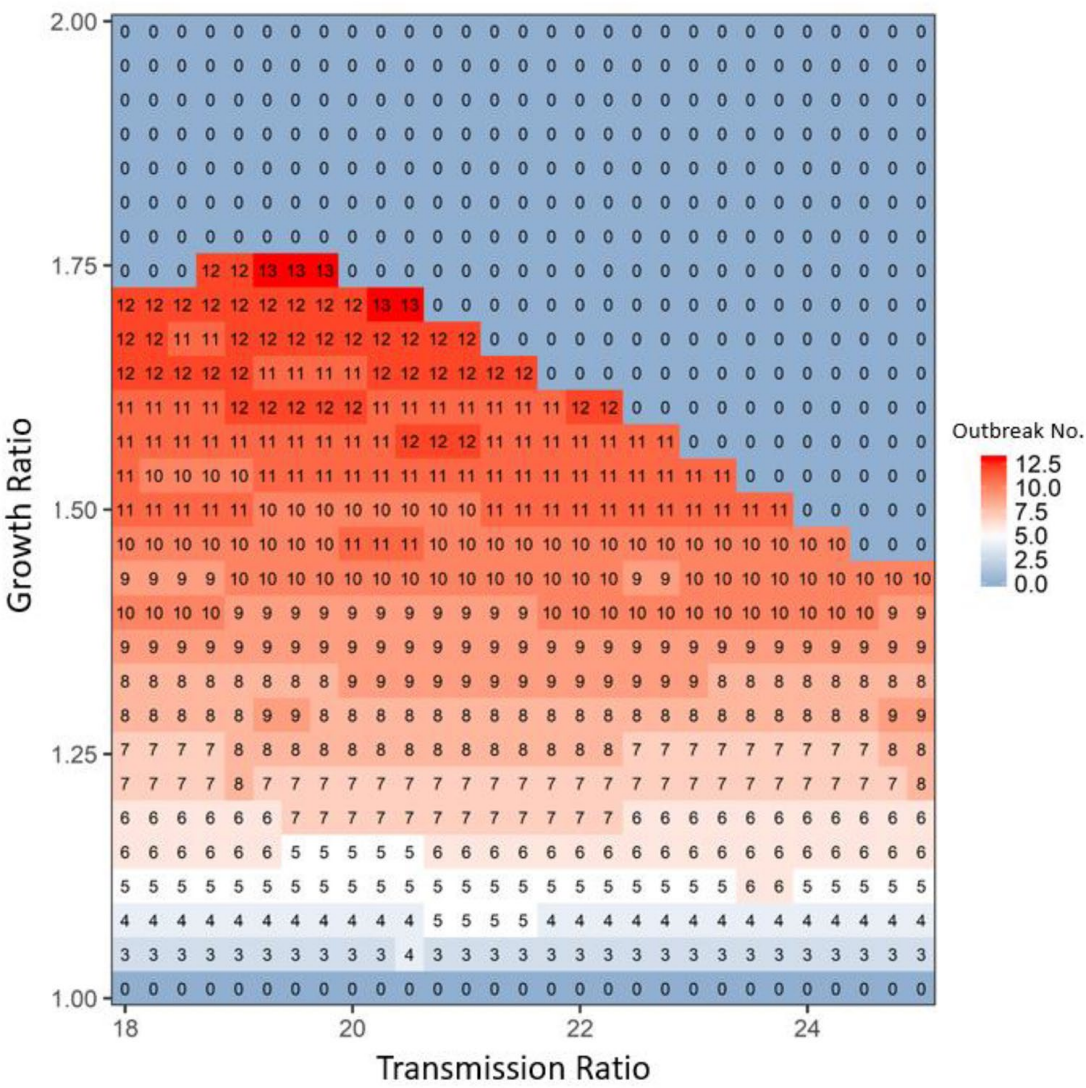
The average disease outbreak numbers in the last 25 generations (total simulated generations are 50) under the influences of growth ratio and transmission ratio for Wild type at patch #30, where robust type provides the baseline growth rate and transmission rate. The color trend with numbers represent the trend of disease outbreak numbers: from blue to white to red, outbreak numbers increase. All the other parameters are: *r*_*R*_ = 0.15, *r*_*Wd*_ = *r*_*Rd*_ = 0.01, *r*_*Wr*_ = *r*_*Rr*_ = 0.2, α_*W*_ = α_*R*_ = 0.05, μ_*W*_ = μ_*R*_ = 0.0005, γ_*W*_ = γ_*R*_ = 0.05, β_*RR*_ = β_*WR*_ = 0.000005 and *m*(*k*,*j*) = 0.000008. In focal patch #1, the initial susceptibles of both types are 200, infecteds are 5, and recovereds are 0. Other patches show similar patterns.

**Figure 4:**
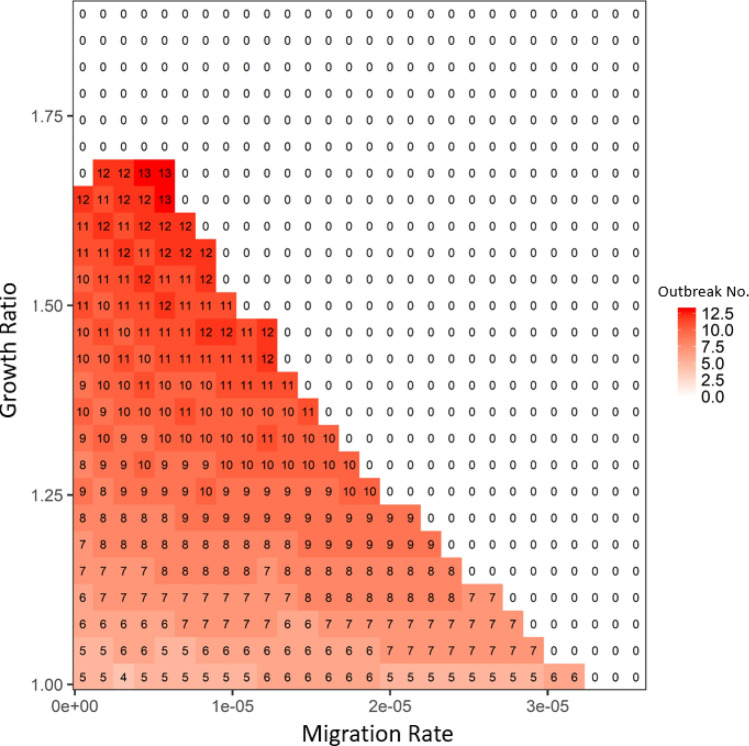
The average disease outbreak numbers in the last 25 generations (total simulated generations are 50) under the influences of growth ratio and migration rate for Robust type at patch #30, where robust type provides the baseline growth rate (in the absence of infection: i.e., *rR*). The color trend with numbers represent the trend of outbreak numbers: from blue to white to red, outbreak numbers increase. All the other parameters are: *r*_*R*_ = 0.15, *r*_*Wd*_ = *r*_*Rd*_ = 0.01, *r*_*Wr*_ = *r*_*Rr*_ = 0.2, α_*W*_ = α_*R*_ = 0.05, μ_*W*_ = μ_*R*_ = 0.0005, γ_*W*_ = γ_*R*_ = 0.05, β_*RR*_ = β_*WR*_ = 0.000005 and β_*RW*_ = β_*WW*_ = 0.0001. In focal patch #1, the initial susceptibles of both types are 200, infecteds are 5, and recovereds are 0. Other patches show similar patterns.

By increasing the competition of the wild type (i.e., the apparent competition with robust-type host via sharing one type of pathogen), the growth ratio would shorten the time for the wild type to increase (if the system had repeated disease outbreaks); thus, the time for one period of disease outbreak could be reduced (compare the breaths of outbreaks in Fig. 1e,f), i.e., the number of outbreaks would be expected to increase, given 25 generations (see the increase in the number of outbreak cycles with the increase of the growth ratio under the bifurcation line in Figs. 3 and 4). Once the growth ratio was large enough to benefit the robust type, the robust type would gradually increase to dominate the system, leading to the disappearance of periodical disease outbreaks (see the 0 outbreak cycles above the bifurcation line in Figs. 3 and 4).

Unlike the dual possible effects of the growth ratio, the transmission ratio always increased the disease prevalence and therefore benefited the robust type (see the color trends along the x-axis in Figs. 2and Fig. S5 in Appendix 2). This unidirectional selection favoring the robust type gradually led the robust type to dominate the entire system, decreasing the possibility of disease periodical outbreaks (see the general decrease in outbreak numbers along x-axis in Fig. 3). When the transmission ratio was relatively small, its benefit to the robust type was concomitantly small; thus, the benefit of the growth ratio to the wild type was easily seen, leading to a faster increase in wild-type density, even with a lower growth ratio (see the lower bifurcation line when transmission ratio was less than 19 in Fig. 3). In contrast, when the transmission ratio was large, the disease prevalence increased (compare the three lines in Fig. 1e,f). In this case, a small increase in the growth ratio could again increase prevalence, in which case selection would favor the robust type (see the lower bifurcation line when transmission ratio was larger than 20 in Fig. 3). Therefore, an intermediate transmission ratio (see the vertical lines near transmission ratio as 19.5 in Fig. 3) would have the highest growth ratio to exhibit disease-driven selection on robust type, leading to a unimodal pattern in the bifurcation line along the transmission ratio (see the trend of the bifurcation line along x-axis in Fig. 3 and Fig. S5 in Appendix 2).

**Figure 5:**
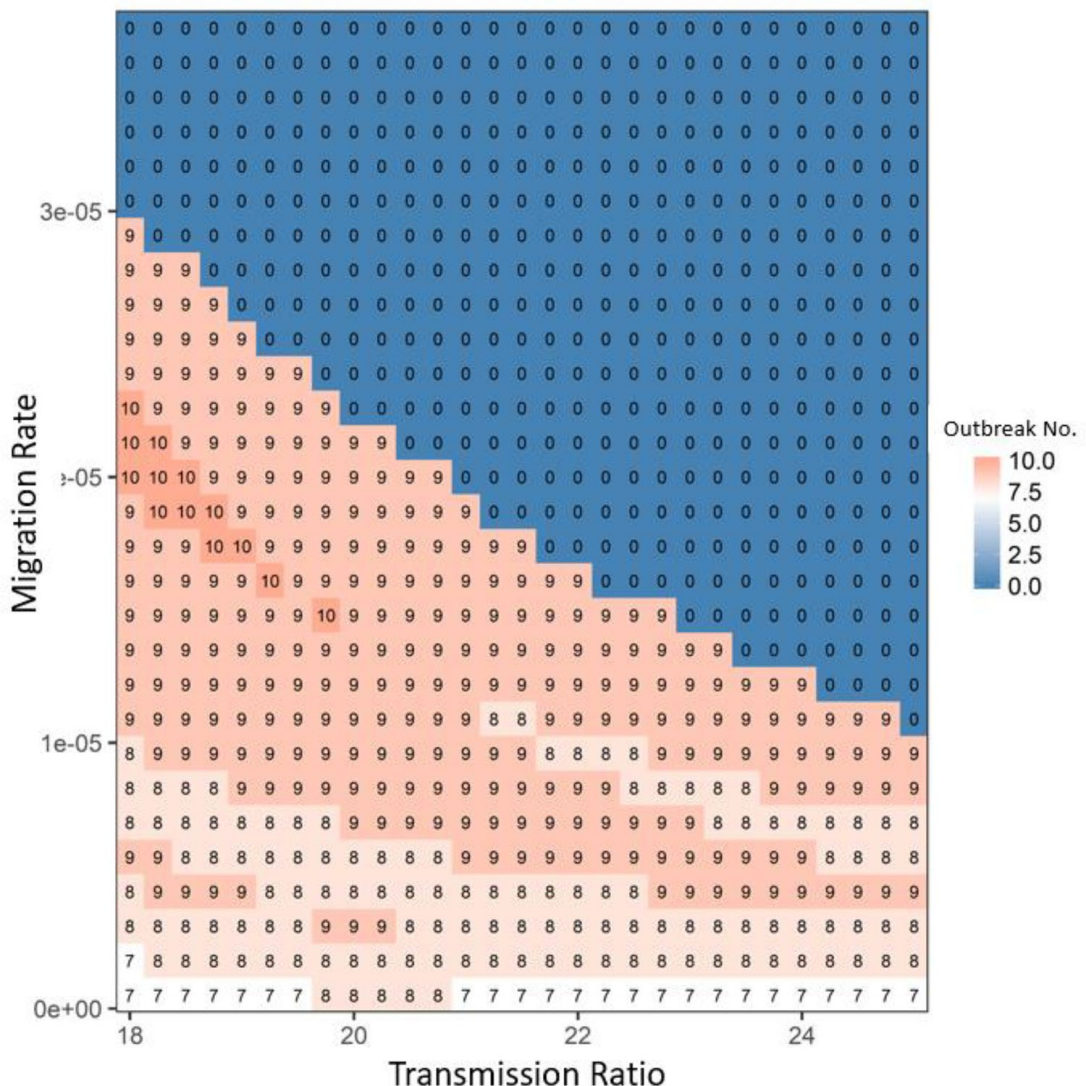
The average disease outbreak numbers in the last 25 generations (total simulated generations are 50) under the influences of transmission ratio and migration rate for patch #30, where robust type provides the baseline transmission rate. The color trend with numbers represent the trend of outbreak numbers: from blue to white to red, outbreak numbers increase. All the other parameters are: *r*_*R*_ = 0.15, *r*_*W*_ = 0.2, *r*_*Wd*_ = *r*_*Rd*_ = 0.01, *r*_*Wr*_ = *r*_*Rr*_ = 0.2, α_*W*_ = α_*R*_ = 0.05, μ_*W*_ = μ_*R*_ = 0.0005, γ_*W*_ = γ_*R*_ = 0.05 and β_*RR*_ = β_*WR*_ = 0.000005. In focal patch #1, the initial susceptibles of both types are 200, infecteds are 5, and recovereds are 0. Other patches show similar patterns.

The migration rate had a similar function as the transmission ratio: the increase in migration rate increased spreading of disease throughout the host metapopulation structure and thereby increased the selective advantage of the robust type (see the increase trend along x-axis in Fig. S4 in Appendix 2). This advantage would decrease the chance of disease periodical outbreaks in the system (see the decrease of outbreak number along x-axis in Fig. 4). Similar to the transmission ratio, the selective advantage of the robust type from the growth ratio also exhibited a unimodal pattern determined by the migration rate (see the trend of the bifurcation line along migration in Fig. S4 in Appendix 2). The only difference was that once the growth ratio was sufficiently large, almost every patch showed a strong selective advantage for the robust type, which led to the synchrony in the metapopulation. In that case, disease overtook the entire system without depending on migration (densities of both host types did not change with migration once growth ratio was above the bifurcation line in Fig. S4 in Appendix 2). Therefore, migration and transmission had interchangeable roles on disease-driven selection in the entire system: i.e., once the migration rate was large, the system only needed a small transmission ratio to achieve the dominance of robust type (see the general 1–1 line in Figs. 5 and S5 in Appendix 2). Decreasing the total number of patches in the system would be equivalent to increasing the migration rate (i.e., shorter pathway leads to faster disease transmission among patches give the same migration rate), which could reinforce the disease and lead to the dominance of robust-type host (see Fig. S6 in Appendix 3). In contrast, increasing total patch number would slow down disease transmission and may cause the disease to die out and be lost to the system during migration (see Fig. S7 in Appendix 3).

### Patch structures with more edges

**Figure 6:**
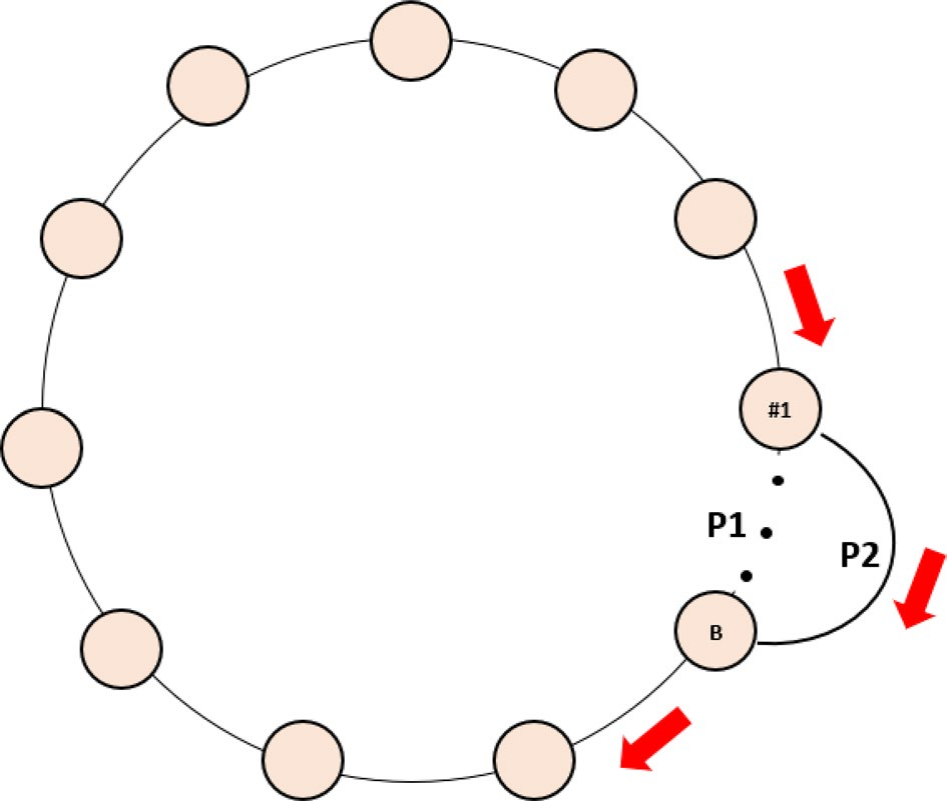
The stepping-stone host metapopulation structure with two ends wrapped around and one extra edge added between Patch #1 and B where patch #1 is the focal patch where disease is initiated. P1 represents the original pathway for disease transmission, while P2 is the pathway though the added edge. All the circles represent patches, the red arrow show the direction of disease transmission among patches.

By adding one more edge to the stepping-stone metapopulation structure (see Fig. 6), there are then two pathways for disease transmission to occur among patches (i.e., P1 and P2 in Fig. 6). When the initial distance between the two patches connected by the extra edge was small (e.g., 1 or 2 patches in between with a total of 40 patches in our simulation), the two pathways almost overlapped; thus, the disease transmission pattern would not be qualitatively changed in the presence of the extra edge (compare Figa. S3 with S3 in Appendix 1 and 3). When the patch distance was intermediate (e.g., 14 or 19 patches apart out of a total 40 patches in our simulation), there was a slight time-delay in the disease transmission between the two pathways. This delay could reinforce disease spreading across all patches (i.e., disease (re)introduction happened before local disease died out across patches) and selected for robust-type host (see disease dynamics in Fig. S9 in Appendix 3). When the patch distance between A and B was relatively large (e.g., 27 or 30 patches apart out of a total 40 patches), disease along pathway P2, which contained fewer patches than pathway P1, would be more likely to reinforce the infection spread (due to the (re)introduction of infected hosts before local disease died out) and lead to the dominance of robust host type in the patches along pathway P2 (see the disease dynamics of Patch #1 and #40 in Fig. S10 in Appendix 3). Therefore, when disease from P1 passed through those patches in P2, the disease was more likely to be lost from the system (due to the greater abundance of robust-type hosts decreasing disease success), leading to the dominance of wild-type hosts in those patches solely on the pathway P1 (see the disease dynamics of Patch #15 and #25 in Fig. S10 in Appendix 3). Similar patterns held as even more edges were added to the system (e.g., two extra edges from focal patch to near neighbor patches; see Fig. S11 in Appendix 3). In general, the more edges were added to the system, the more connected the system would be (e.g., a well-connected system acts like one big patch), which would facilitate disease transmission; thus, making it more likely for robust-type hosts to dominate the system. This result is consistent with the previous study for a non-spatial host system^35^.

## Discussion

Epidemiological models of populations facing novel, highly contagious, deadly infections generally predict poor chances for positive population growth^20,49–52^. Evolutionary rescue models for such populations have offered better hopes of persistence from the intense selective pressure of a single, sudden outbreak of a novel pathogen, so long as genes permitting robustness in the face of infection are sufficiently represented in the affected population^1,10,53,54^. Here, we considered the murky middle ground in which novel pathogens provide an inconstant selective pressure as ecological, epidemiological, and host evolutionary dynamics all interact with each other over time.

Within any metapopulation facing a novel infectious threat, we see the logical possibility for many different outcomes. A severe enough initial outbreak in a population can drive local host extinction, thereby also eliminating the disease from the entire metapopulation system. On the other extreme, there are at least two distinct paths that could lead to the natural eradication of the pathogen while the host population persists. The first is an evolutionary rescue scenario in which the protective genes reduce host susceptibility to infection, such that after infecting and killing off the wildtype, the disease cannot successfully infect the (potentially few) remaining robust hosts^10,53,54^ (Similar as Fig. 1b,d and f; Fig. S1 in Appendix 1). The second is a herd immunity scenario, in which all remaining hosts (whether wildtype or robust) are the (potentially few) survivors of infection and are now immune, leaving no new susceptible hosts for the disease to infect^55–57^ (Similar as the one-patch case in Fig. 1a,c,e; Fig. S2 in Appendix 1). In both scenarios, the population could potentially recover once the disease has been eradicated, however, there are critical differences in how they might be affected by the reintroduction of the disease (see below).

Of course, in either of these cases, the pressures may be insufficient to eradicate the disease, but may lead instead to steady endemicity. In the case of evolutionary rescue, protective genotypes may also incur other fitness trade-offs leading to the maintenance of the wildtype genes in the population, allowing ongoing circulation of infection, but at insufficiently large scales to wipe out the remaining wildtype population^10^. In the case of herd immunity, birth rates, immigration, and gradual loss of immune memory may replenish the susceptible host population quickly enough to allow the reproductive number for the disease to hover near one, neither allowing for large outbreaks nor eradicating infection completely^58,59^. In both cases, we would expect host population persistence, but at decreased size/growth rates than had been seen prior to the introduction of infection.

Lastly, and most interestingly, there are scenarios in which the epidemiological and evolutionary dynamics go through cycles (Figs. 2, 3 and 4 and Fig. S3 in Appendix 1). In the case of evolutionary rescue, disease would only have large-scale impacts on the population once the percent of the population with the robust genotype had decreased sufficiently, which is most likely in the case of eradication followed by reintroduction (though could be achieved cycling between endemic and epidemic conditions based on gene frequencies). Increasing wildtype representation after initial evolutionary rescue could be due to either to genetic drift or else (and potentially more rapidly) to alternative selective pressures that acted on the population in the absence of the disease. If the population’s wildtype had recovered to be the majority, reintroduction after eradication could start the cycle anew (Note that we do not mean to imply these cycles would be stable, merely that we would expect temporal cycles in outbreaks, leading to rapid evolutionary rescue, leading to local decrease in the intensity of the selective pressure, leading to the potential recovery of the wildtype in the population, allowing for a new outbreak).

In the case of herd immunity, protection can be lost much faster as gradual loss of immune memory, immigration, and births lead to the replenishment of the susceptible portion of the population^60^. Once a sufficient percent of the population is again susceptible, reintroduction would be expected to lead to another large-scale outbreak based on the effective reproductive number of the disease^61^. Of course, herd immunity may not have prevented some evolutionary outcomes from the selective pressure of the disease, meaning that each cycle might take longer and longer to replenish to allow for the next outbreak, since each cycle might slightly increase the percent representation of the robust type in the population, however, the dynamics of epidemiological and evolutionary factors will act in synergy to dictate the frequency and impact of such outbreaks.

Of course, in each of the cases studied, we have allowed evolutionary dynamics only in the host. For conservation purposes, a host system with novel disease (see White Nose Syndrome in bats^1^) could be very common^10^. Hence, the cases with evolved host but constant pathogen should be more explored in future. Our goal of this paper is to study this simplified scenario to better understand its already complicated dynamics as a first step in understanding and predicting the host evolution and even coevolutionary potential of these systems. Future work will explore each of these cases when the pathogen is also allowed to evolve in response to host pressures. Also, here we assumed a simple clockwise migration direction among patches as an extension of the "stepping-stone" model and also explored the potential for more complicated spatial structure by adding more edges to the “stepping-stone” structure. While we do not expect this is a realistic migration structure, we did this to highlight the potential for these evolutionary dynamics. Other ways of migration^10,17,62–64^ (e.g., full random or differential movement due to patch heterogeneity) combined with certain metapopulation structures may change the cycling patterns in this study. In this study, we also generally chose parameters to only generate one cycle of disease outbreak in the absence of migration. Future work can further explore the parameter space that could lead to disease periodical outbreaks without migration^65,66^ (e.g., demographic process or seasonality, or demographic stochasticity).

Critically, our model has also shown that monitoring any one feature from metapopulation dynamics, shifting selective pressures, and disease transmission to understand the impact of disease introduction may give a woefully insufficient understanding of the viability of the population over the longer term. Heroic interventions may be unnecessary if the driving dynamic early after introduction is (successful) evolutionary rescue^1^ but may be critical if herd immunity is likely to curtail the full selective impact of evolutionary rescue dynamics. If metapopulation dynamics lead to host movement on a slow enough timescale to allow for disease persistence in the system, but gradual local eradication of disease from any one patch, epidemiological monitoring may lead to premature cessation of management efforts. In these cases, it may actually be that increasing natural rates of host movement among patches, or even purposeful disease introduction to synchronize disease dynamics across all patches could benefit the long-term probability of host persistence (so long as truly external reintroduction of infection was unlikely). Overall, our model has allowed us to characterize the relative impact of epidemiological, ecological, and evolutionary dynamics in a metapopulation structure to explore how each contributes to shaping the probabilities of pathogen and host persistence. Especially as disease surveillance technologies^67–69^, including genetic sequencing, become more easily and affordably available, we believe this eco-evo-epi framework will be critical in generating concrete hypotheses for empirical testing and allow us a much fuller understanding of how disease risks affect the natural world.

## Supplementary Information

Supplementary Information.
