# Supplementary material for: The dynamics of evolutionary rescue from a novel pathogen threat in a host metapopulation
Source: Sci Rep. 2021 May 25;11:10932. doi: 10.1038/s41598-021-90407-z (PMC8149858; doi:10.1038/s41598-021-90407-z)
Supplement: Supplementary file 1 — Supplementary Information. [file 41598_2021_90407_MOESM1_ESM.pdf]

Appendices for the following manuscript:

Title: The Dynamics of Evolutionary Rescue from a Novel Pathogen Threat in a Host Metapopulation

Running Head: Eco-Evo-Epidemiological Dynamics

Authors: Jing Jiao*[1, 2], Nina Fefferman[1, 3]

Institutions and emails:

[1]National Institute for Mathematical and Biological Synthesis, The University of Tennessee, 1122 Volunteer Blvd., Suite 106, Knoxville, Tennessee 37996 USA; Email: jjiao3@utk.edu

[2]Department of Biological Science, Florida State University, 319 Stadium Dr, Tallahassee, FL 32304 USA

[3]Ecology & Evolutionary Biology, The University of Tennessee, 1416 Circle Drive, Knoxville, Tennessee 37996 USA; Emails: nina.h.fefferman@gmail.com

Appendix 1 The dynamics of Susceptible (S), Infected (I) and Recovery (R) in four patches under the stepping-stone host metapopulation structure

Under the stepping-stone host metapopulation structure (two ends were wrapped around; see Fig. 1) with total 40 patches, the dynamics of Susceptible (S), Infected (I) and Recovery (R) individuals in all patches were simulated based on Latin Hypercube sampling (LHS; see Kollig and Keller 2002) of three factors: growth ratio of wild type vs. robust (robust growth rate was fixed), transmission ratio of wild type vs. robust (robust transmission rate was fixed) and migration rate (both types across S, I, R stages were assumed to have the same migration rate). Based on the results of LHS, the S, I, R dynamics showed the following three types of patterns (i.e., the following three scenarios) in all 40 patches. Here only four patches' dynamics (Patch #1, #15, #30 and #40) were showed in all the following figures, but other patches exhibit similar patterns.

Here we also calculated the basic reproductive number R_0 in the presence of migration to better understand the following three scenarios. Here we inferred R_0 at one special case when migration term m_{ji}^{I} in Eq. 2 has linear relationship with I_{ji} (i.e., $m_{ji}^{I} = \rho I_{ji}$ for both genotypes):

$$R_0 = \frac{\beta_W B_{jW|I=0} + \beta_R B_{jR|I=0}}{\mu(\alpha+\mu+\gamma-\rho)} \quad (20)$$

where $B_{jW|I=0}$ and $B_{jR|I=0}$ are reproduction rate of each genotype (*W* or *R*) in the absence of disease. Under our parameter setup, disease in one patch would die out without migration (i.e., $R_0 < 1$ in the absence of migration; see Fig. 2). By adding migration, R_0 could change from Eq. 19 to Eq. 20 when immigration arrived before disease died out. Although Eq. 20 is only for a special case, the general conclusion from Eq. 20 can still provide insights for other situations.

We could also deduce effective reproductive number R:

$$R = \frac{\beta_W S_W + \beta_R S_R}{\alpha + \mu + \gamma} \tag{21}$$

Where S_W and S_R changed with time, so R is also time dependent.

Scenario 1: *robust type dominated the system*

If the difference in transmission rates of the two host genotypes was high (i.e., β_W is large given a fixed β_R), robust type would dominate the system in the presence of disease (see Fig. S1), which leads to the increase of R_0 (see Eq. 20) and a relatively larger R in Fig. S1. This situation is not good at disease spreading.

Scenario 2*: wild type dominated the system*

A relatively higher growth rate in wild type could increase wild-type competition and increase the proportion of wild type in host population (see the dominance of wild type in Fig. S2). Wild type is easier to spread the disease than robust type, leading to a lower R in Fig. 2 compared to *Scenario 1*.

Scenario 3: *wild and robust alternated to dominate the system, creating disease cycles*

At certain intermediate parameter space of the above two cases, both host genotypes can coexist in the long run with the periodic switching for the dominance (i.e., when disease was on through migration, robust type increased and dominated the system; when disease was off, wild type could increase and take over the system). This process would create dynamic cycles, leading to the fluctuation of R around 1 (see Fig. S3).

References: Kollig, T. and Keller, A., 2002, September. Efficient multidimensional sampling. In Computer Graphics Forum (Vol. 21, No. 3, pp. 557-563). Oxford, UK: Blackwell Publishing, Inc.

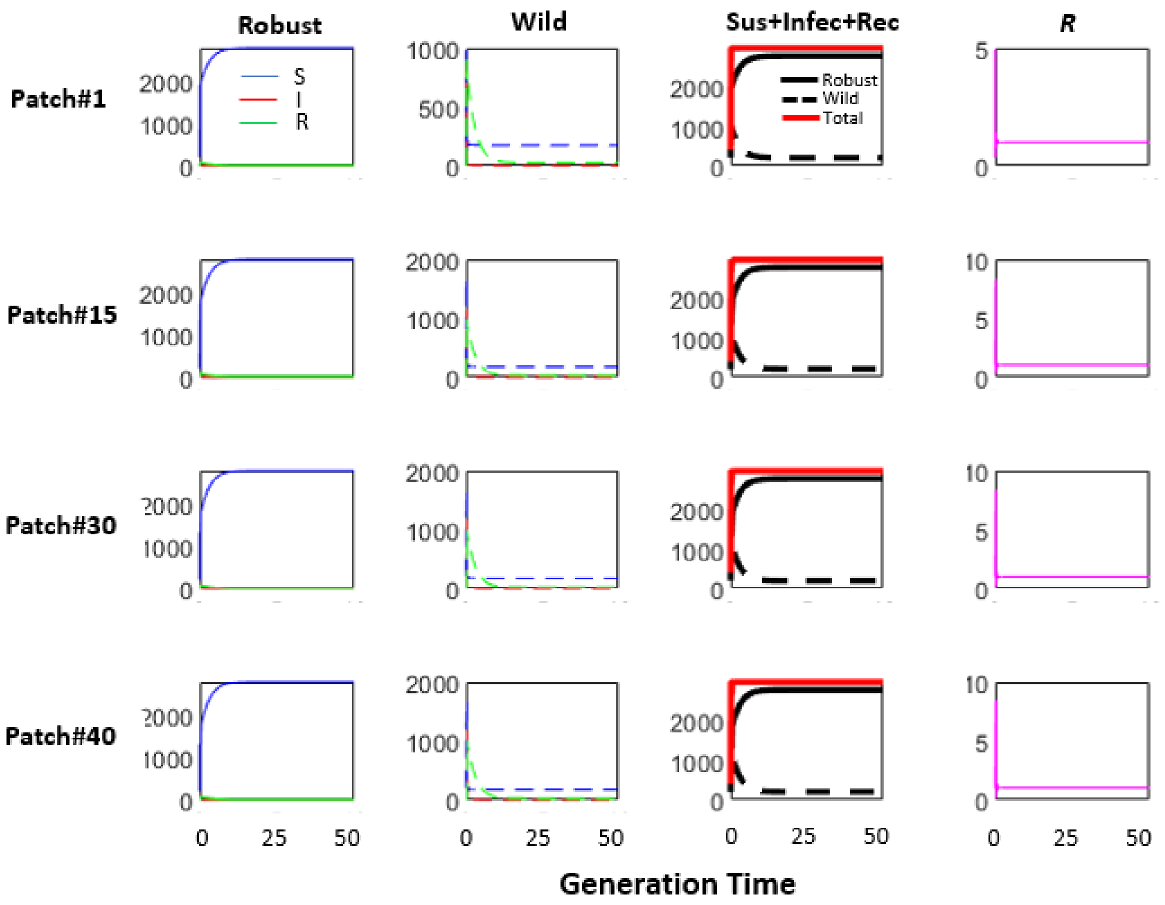

Fig. S1 The dynamics of Susceptibles, Infected, Recovered in robust type and wild type, population size in wild type, robust and total host and effective reproductive number *R* in four patches labelled #1, #15, #30 and #40. S is blue color, I is in red, R is in green with solid in robust and dashed in wild type. All the parameters are: $r_R = 0.16$, $r_W = 0.2$, $r_{Wd} = r_{Rd} = 0.01$, $r_{Wr} = r_{Rr} = 0.2$, $\alpha_W = \alpha_R = 0.05$, $\mu_W = \mu_R = 0.0005$, $\gamma_W = \gamma_R = 0.05$, $\beta_{RR} = \beta_{WR} = 0.000005$, $\beta_{RW} = \beta_{WW} = 0.0005$ and $mig(k, j) = 0.00001$. Initial susceptibles of both types are 200 in each patch, initial infected number of each host type is 5 in #1 patch where disease starts.

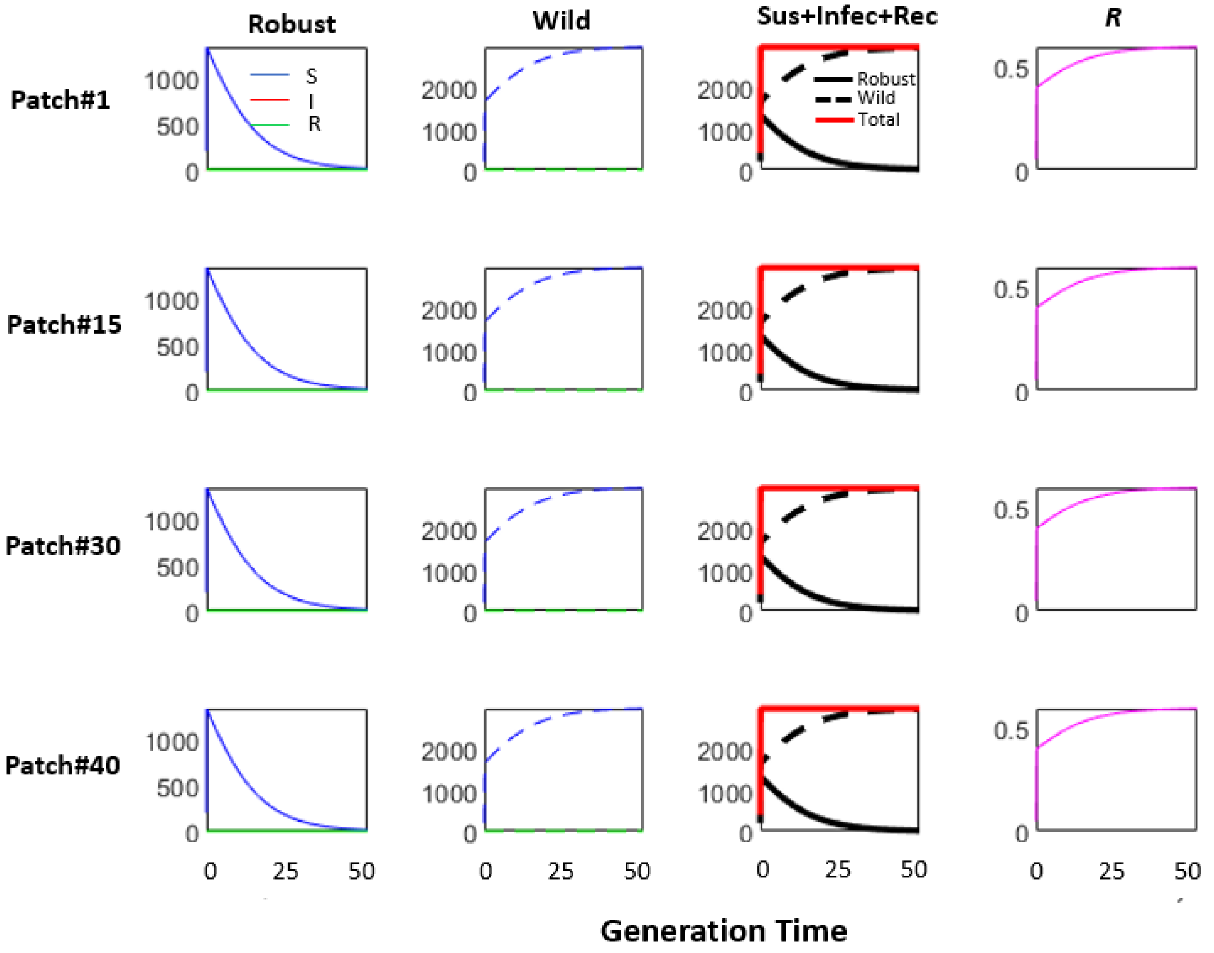

Fig. S2 The dynamics of Susceptibles, Infected, Recovered in robust type and wild type, population size in wild type, robust and total host and effective reproductive number *R* in four patches labelled #1, #15, #30 and #40. S is blue color, I is in red, R is in green with solid in robust and dashed in wild type. All the parameters are: $r_R = 0.16$, $r_W = 0.2$, $r_{Wd} = r_{Rd} = 0.01$, $r_{Wr} = r_{Rr} = 0.2$, $\alpha_W = \alpha_R = 0.05$, $\mu_W = \mu_R = 0.0005$, $\gamma_W = \gamma_R = 0.05$, $\beta_{RR} = \beta_{WR} = 0.000005$, $\beta_{RW} = \beta_{WW} = 0.00002$ and $mig(k,j) = 0.00001$. Initial susceptibles of both types are 200 in each patch, initial infected number of each host type is 5 in #1 patch where disease starts.

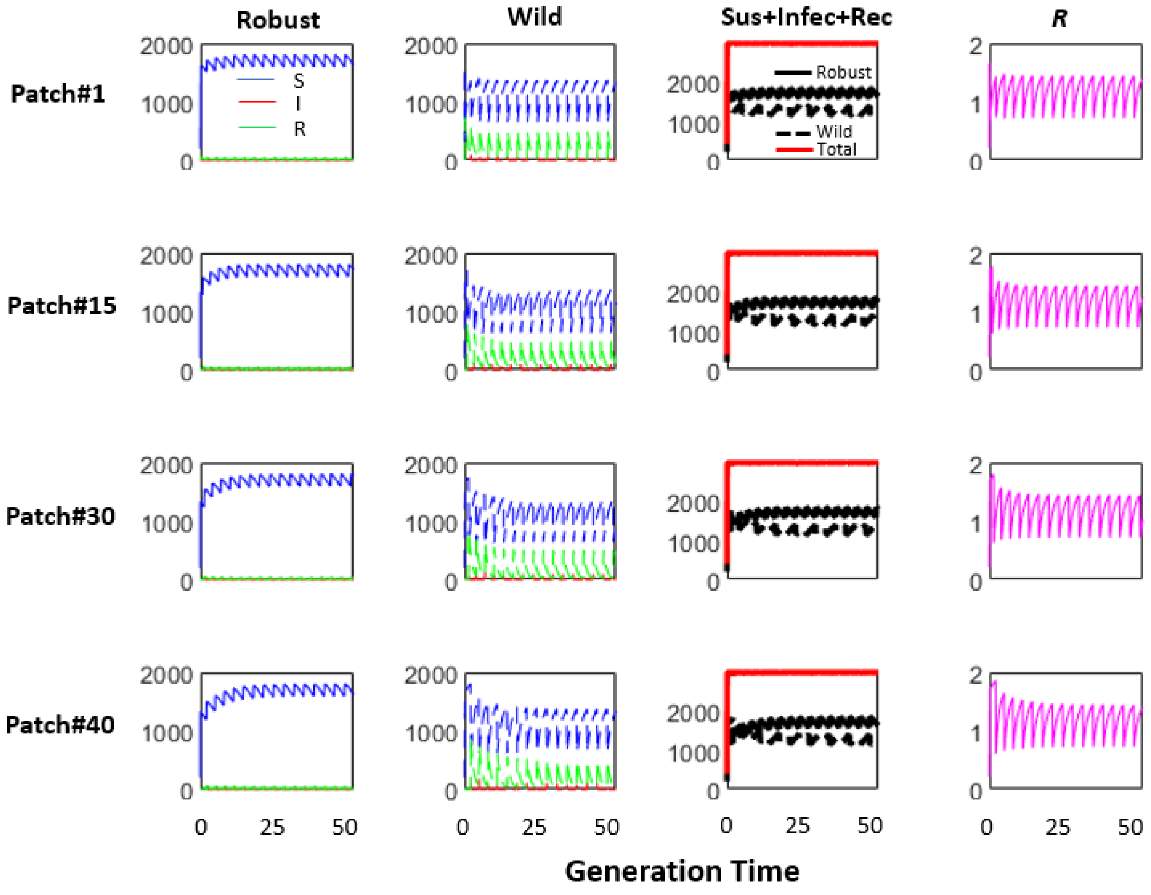

Fig. S3 The dynamics of Susceptibles, Infected, Recovered in robust type and wild type, population size in wild type, robust and total host, and effective reproductive number *R* in four patches labelled #1, #15, #30 and #40. S is blue color, I is in red, R is in green with solid in robust and dashed in wild type. All the parameters are: $r_R = 0.16$, $r_W = 0.2$, $r_{Wd} = r_{Rd} = 0.01$, $r_{Wr} = r_{Rr} = 0.2$, $\alpha_W = \alpha_R = 0.05$, $\mu_W = \mu_R = 0.0005$, $\gamma_W = \gamma_R = 0.05$, $\beta_{RR} = \beta_{WR} = 0.000005$, $\beta_{RW} = \beta_{WW} = 0.0001$ and $mig(k,j) = 0.00001$. Initial susceptibles of both types are 200 in each patch, initial infected number of each host type is 5 in #1 patch where disease starts.

Appendix 2 The influences of growth ratio vs. migration rate, transmission ratio vs. migration rate on the numbers of host genotypes

When transmission ratio was fixed as 20, we simulated the average numbers of the two host genotypes in the last 25 generations under the influences of growth ratio vs. migration rate, transmission ratio vs. migration rate.

Similar as Fig. 3, the growth ratio showed dual opposite functions: the increase of growth ratio would first increase the competition of wild type, leading to an increase in the number of wild type (see the red color trend with the increase of growth ratio in the left panel of Fig. S4). Once growth ratio reached certain level, the susceptible individuals would increase, leading to an increase in disease and the number of robust but decrease in wild type (see the color change across the bifurcation lines in Fig. S4). Once growth ratio increased in further, the competition of wild type could be observed again (the increase in wild type; see the color trend in the upper left above the bifurcation line in Fig. S4).

Increased migration rate increased the spread of disease, leading to an increase in the number of robust types. However, when migration was large enough (see the bottom right of the bifurcation line in Fig. S4), the disease could spread across the entire system quickly. In that case, further increases in migration rates would be not necessary for disease spread; hence, the numbers of both host types were less sensitive to large migration rates (see the less pronounced change on the bottom right in Fig. S4). Once growth ratio was large, disease existed in almost all patches (due to the increased susceptible individuals) even in the absence of migration. In this situation, robust type would dominate the system easily and showed less sensitivity towards the increase of migration rate (see the areas above the bifurcation lines in Fig. S4).

Both migration rate and transmission ratio would benefit robust type: i.e., migration could increase the chance of disease prevalence in the system, while large transmission ratio would lead to strong disease selection on robust. This synergy led to a tradeoff pattern in shaping the numbers of host genotypes: the same amount of increase in robust type (or the same decrease in wild type) could be achieved by either large migration or large transmission ratio (see the near 1-1 bifurcation line in Fig. S5).

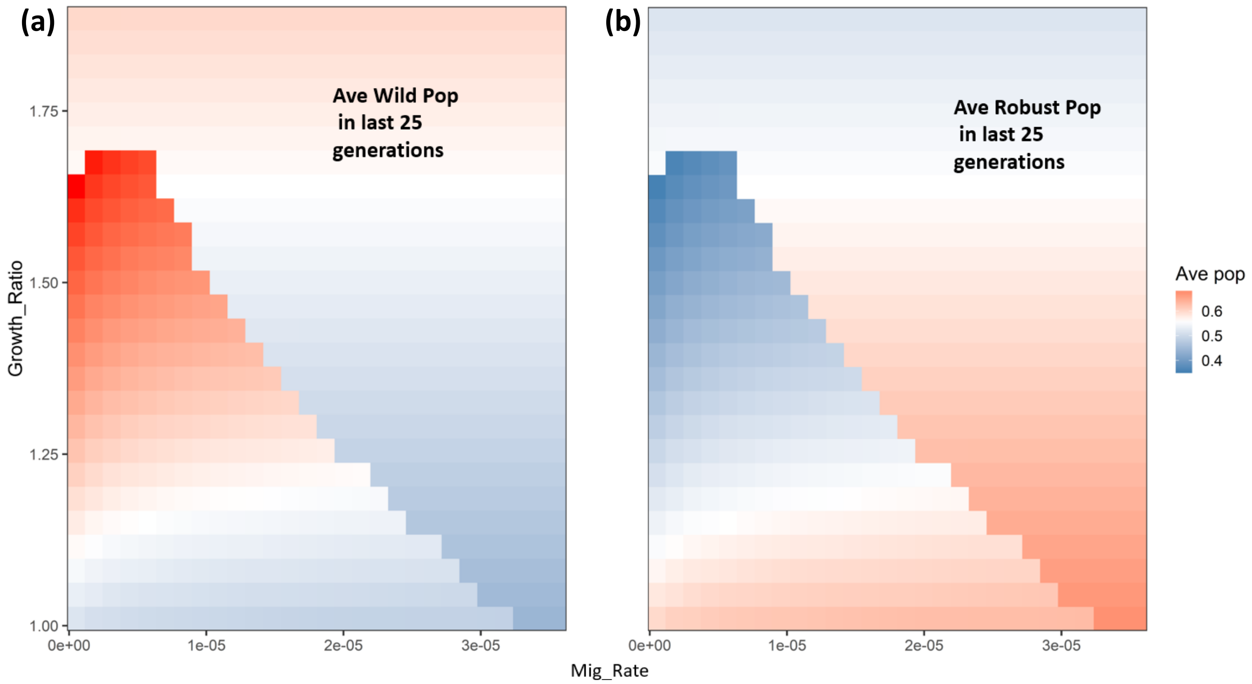

Fig. S4 The standardized average population size (population size divided by carrying capacity) of both wild type and robust type in the last 25 generations (total simulated generations are 50) under the influences of migration rate and growth ratio at patch #30, where robust type provides the baseline growth rate. The color trend represents the average population size: from blue to white to red, average population sizes increase. All the other parameters are: $r_R = 0.15$, $r_{Wd} = r_{Rd} = 0.01$, $r_{Wr} = r_{Rr} = 0.2$, $\alpha_W = \alpha_R = 0.05$, $\mu_W = \mu_R = 0.0005$, $\gamma_W = \gamma_R = 0.05$, $\beta_{RR} = \beta_{WR} = 0.000005$ and $\beta_{RW} = \beta_{WW} = 0.0001$. In focal patch #1, the initial susceptibles of both types are 200, infecteds are 5, and recovereds are 0.

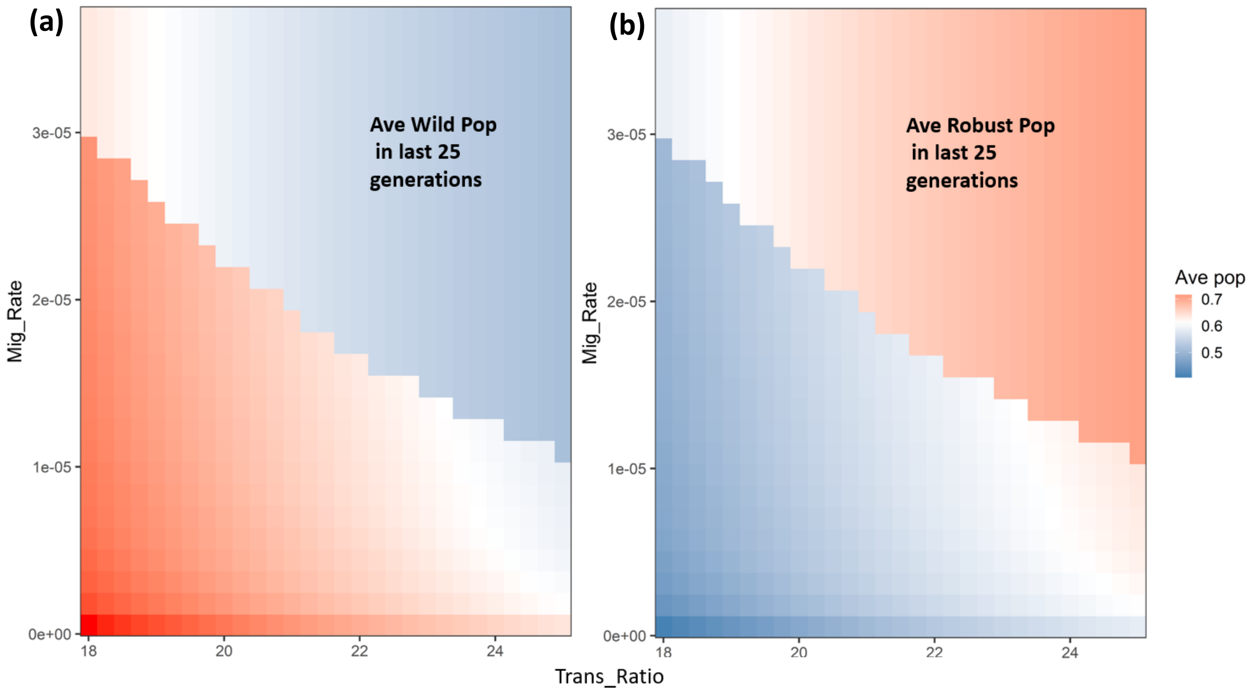

Fig. S5 The standardized average population size (population size divided by carrying capacity) of both wild type and robust type in the last 25 generations (total simulated generations are 50) under the influences of transmission ratio and migration rate at patch #30, where robust type provides the baseline transmission rate. The color trend represents the average population size: from blue to white to red, average population sizes increase. All the other parameters are: $r_R = 0.15$, $r_R = 0.2$, $r_{Wd} = r_{Rd} = 0.01$, $r_{Wr} = r_{Rr} = 0.2$, $\alpha_W = \alpha_R = 0.05$, $\mu_W = \mu_R = 0.0005$, $\gamma_W = \gamma_R = 0.05$ and $\beta_{RR} = \beta_{WR} = 0.000005$. In focal patch #1, the initial susceptibles of both types are 200, infecteds are 5, and recovereds are 0.

Appendix 3 The influences of total patch number and extra edge connections on the disease dynamics in the system

From the above "stepping-stone" metapopulation structure with clockwise host migration among patches, we saw that both the migration rate of host and the patch number influenced the timing of disease arrival in each patch. If disease transfers among patches very quickly (either through higher migration rate or fewer total patches), the new disease could re-arrive at a patch before the previous outbreak had died out, leading to the reinforcement of disease in the system. If disease transmission among patches is very slow (lower migration rate or more total patches), the disease may be lost during migration. Here we further studied how sensitive the disease dynamics are to changes in the total patch number. In addition, we also explored how adding one extra edge to the "stepping-stone" structure (i.e., increasing connectivity) can change the model results.

As we expected, under the parameter setting as Fig. S3, where disease can produce periodical outbreaks in each patch, gradually reducing the total patch number from 40 to 33, leads to the disease transmitting fast enough to reinforce local outbreaks and promotes the dominance of robust-type hosts (see Fig. S6: periodical outbreaks gradually ceased). On the contrary, when we increase the patch number to 190, the disease transmission among patches slows and eventually gets lost during migration (see Fig. S7). Hence, the wild-type host gradually dominates the system once disease is almost eradicated; however, the increased wild-type hosts also provide plenty of susceptibles, therefore, the disease starts to recover. This leads to the dominance of robust-type hosts over sufficient time (see the change in effective R_0 in Fig. 8).

Adding one extra edge to the host “stepping-stone” patch structure would generate two pathways for disease transmission (i.e., P1 and P2 in Fig. 6): the original pathway before adding the extra edge, and the shorter pathway connected through the extra edge. Depending on the path distance between the two edge-connected patches (i.e., the number of patches between focal patch #1 and B in Fig. 6), the potential patterns for disease through the two pathways could be: 1) close to simultaneous introduction of disease via the two paths when the distance between them is small; 2) slightly time-delayed when the distance between them is moderate; 3) largely time-delayed when the number of patches between them is large.

To test the influence of adding one edge, here we picked up one end of the edge as the focal patch, labelled #1 (i.e., where disease is initiate; focal patch #1in Fig. 6) and the other end of the edge as patch B. We tested the disease dynamics at patch #1 (focal patch), #15, #30 and # 40 following the direction of disease transmission where patch B was assigned to #2, #3, #15, #20, #28 and #31, respectively. We chose the parameter setup as Fig. S3 where disease showed periodic cycles in each patch under the initial “stepping-stone” spatial structure.

Based on the above patterns, we grouped the simulation results with regards to the distance between focal patch #1and B. When the distance between focal patch #1and B is small (e.g., Patch B takes #2, #3), disease dynamics in the system were not meaningfully altered: i.e., under parameter setting as Fig. 3, disease still shows cycling pattern over time (Fig. S8). When the distance was intermediate (e.g., when Patch B takes #15, #20), the disease transmission from the two pathways (P1 and P2 in Fig. 6) have a slight time-difference, so disease in all patches would be reinforced (i.e., new infected hosts would arrive at each patch before local disease in that patch dies out) and robust-type hosts (which are selected for under higher disease prevalence) would dominate the entire system (compare the solid and dashed lines in all four

patches in Fig. S9). When the distance is relatively large (e.g., when Patch B takes #28 and #31), the time-delay of disease transmission through the two pathways is very large. The pathway having fewer patches (i.e., P2 in our case in Fig. 6) would have a disease reinforcement (i.e., disease can pass to one patch earlier before local disease dies out due to the shorter pathway), leading to the dominance of robust-type hosts therein. Robust-type hosts in P2 would be less likely to spread disease (due to fewer susceptible individuals), so disease from P1 gets lost here and cannot be passed to those patches solely along P1. This leads to the dominance of wild-type hosts therein due to their higher growth rate in the absence of the disease (see disease dynamics of the middle two rows in Fig. S10).

With two more edges added, if the path distance along those added two edges are small (e.g., 1 and 2 patches from focal patch #1 to #2 and #3; see Fig. S11), the system can still hold the periodic outbreaks as Fig. 3 without any extra edges. When the distance along the added pathways increases, the system is more likely to show the disease reinforcement pattern: i.e., disease would be passed to the patch where the local outbreak persists; thus, robust-type hosts will tend to be selected for across all the patches.

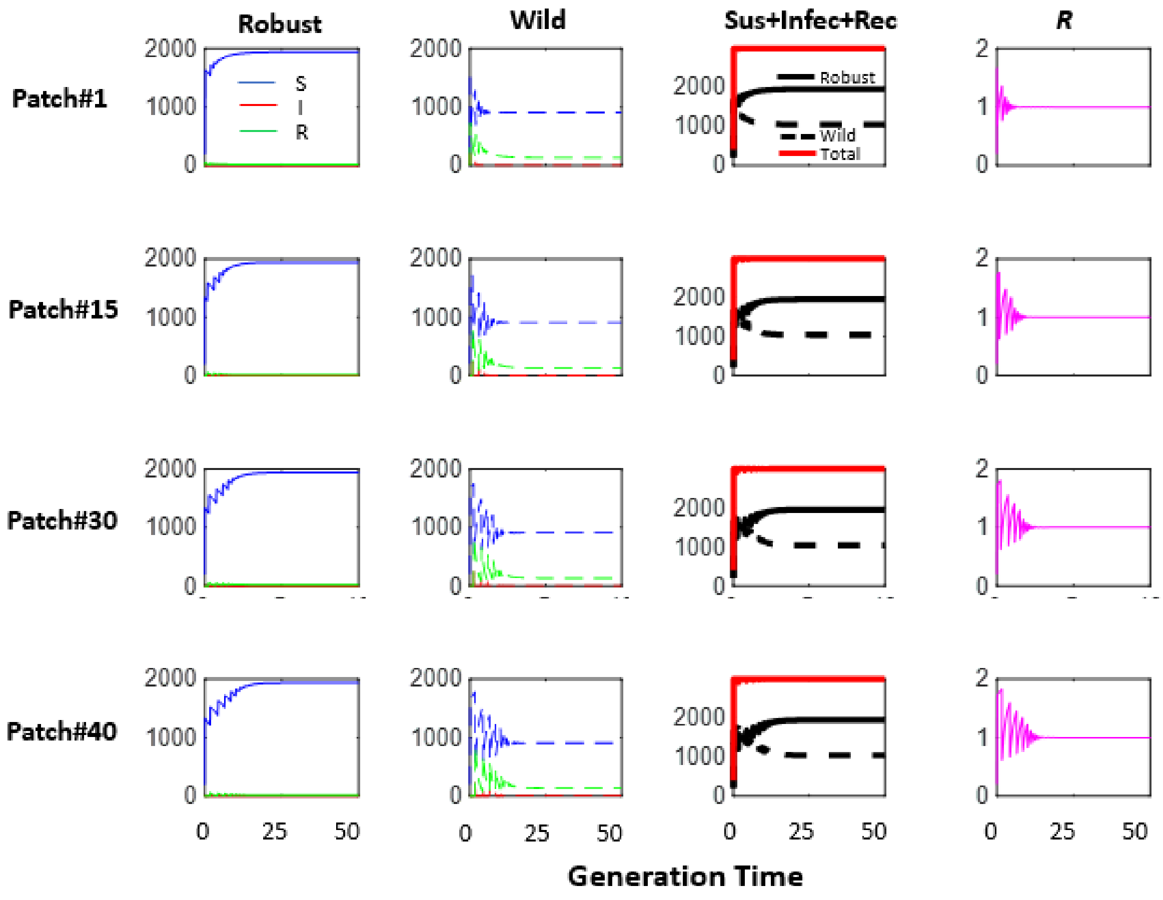

Fig. S6 The dynamics of Susceptibles, Infected, Recovered in robust type and wild type, population size in wild type, robust and total host, and effective reproductive number R in four patches labelled #1, #15, #30 and #40 when total patch number equals to 33. S is blue color, I is in red, R is in green with solid in robust and dashed in wild type. All the parameters are: $r_R = 0.16$, $r_W = 0.2$, $r_{Wd} = r_{Rd} = 0.01$, $r_{Wr} = r_{Rr} = 0.2$, $\alpha_W = \alpha_R = 0.05$, $\mu_W = \mu_R = 0.0005$, $\gamma_W = \gamma_R = 0.05$, $\beta_{RR} = \beta_{WR} = 0.000005$, $\beta_{RW} = \beta_{WW} = 0.0001$ and $mig(k, j) = 0.00001$. Initial susceptibles of both types are 200 in each patch, initial infected number of each host type is 5 in #1 patch where disease starts.

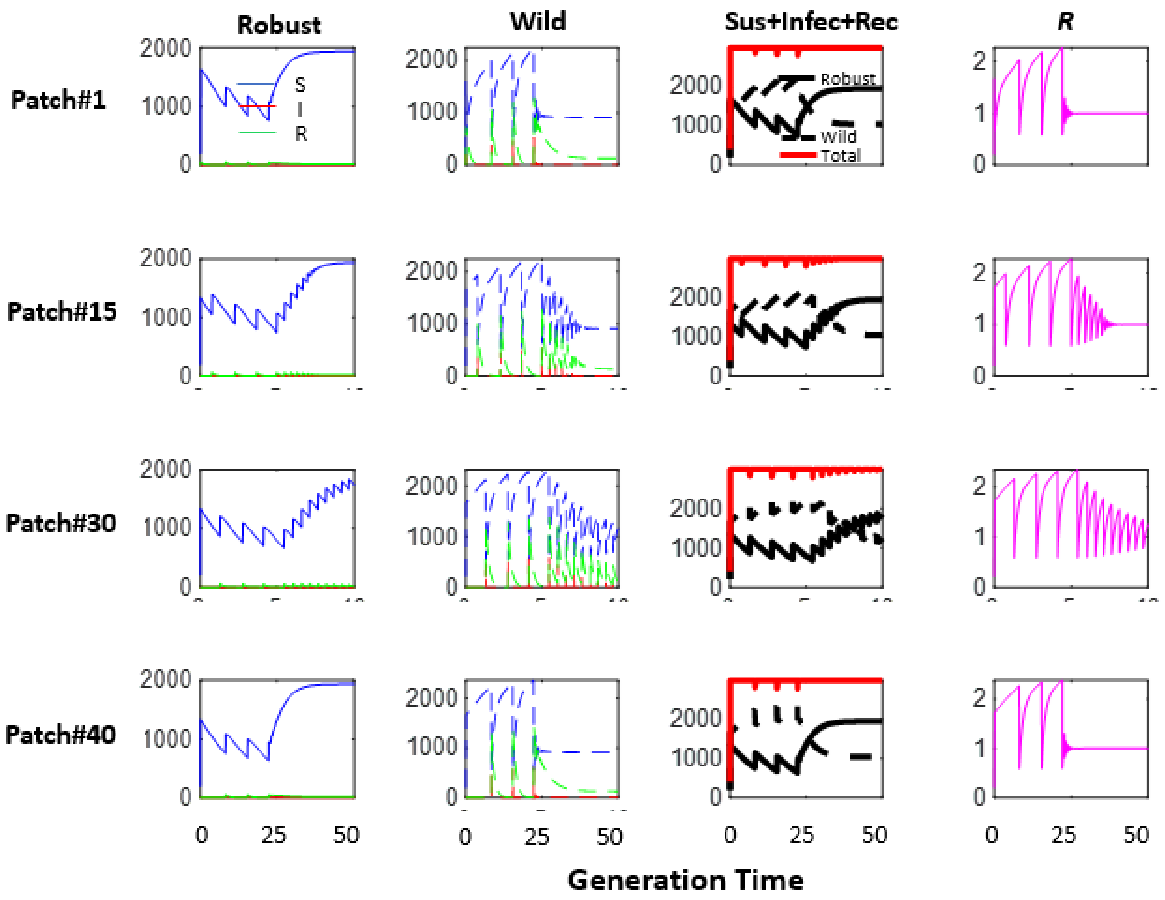

Fig. S7 The dynamics of Susceptibles, Infected, Recovered in robust type and wild type, population size in wild type, robust and total host, and effective reproductive number R in four patches labelled #1, #15, #30 and #40 when total patch number equals to 190. S is blue color, I is in red, R is in green with solid in robust and dashed in wild type. All the parameters are: $r_R = 0.16$, $r_W = 0.2$, $r_{Wd} = r_{Rd} = 0.01$, $r_{Wr} = r_{Rr} = 0.2$, $\alpha_W = \alpha_R = 0.05$, $\mu_W = \mu_R = 0.0005$, $\gamma_W = \gamma_R = 0.05$, $\beta_{RR} = \beta_{WR} = 0.000005$, $\beta_{RW} = \beta_{WW} = 0.0001$ and $mig(k,j) = 0.00001$. Initial susceptibles of both types are 200 in each patch, initial infected number of each host type is 5 in #1 patch where disease starts.

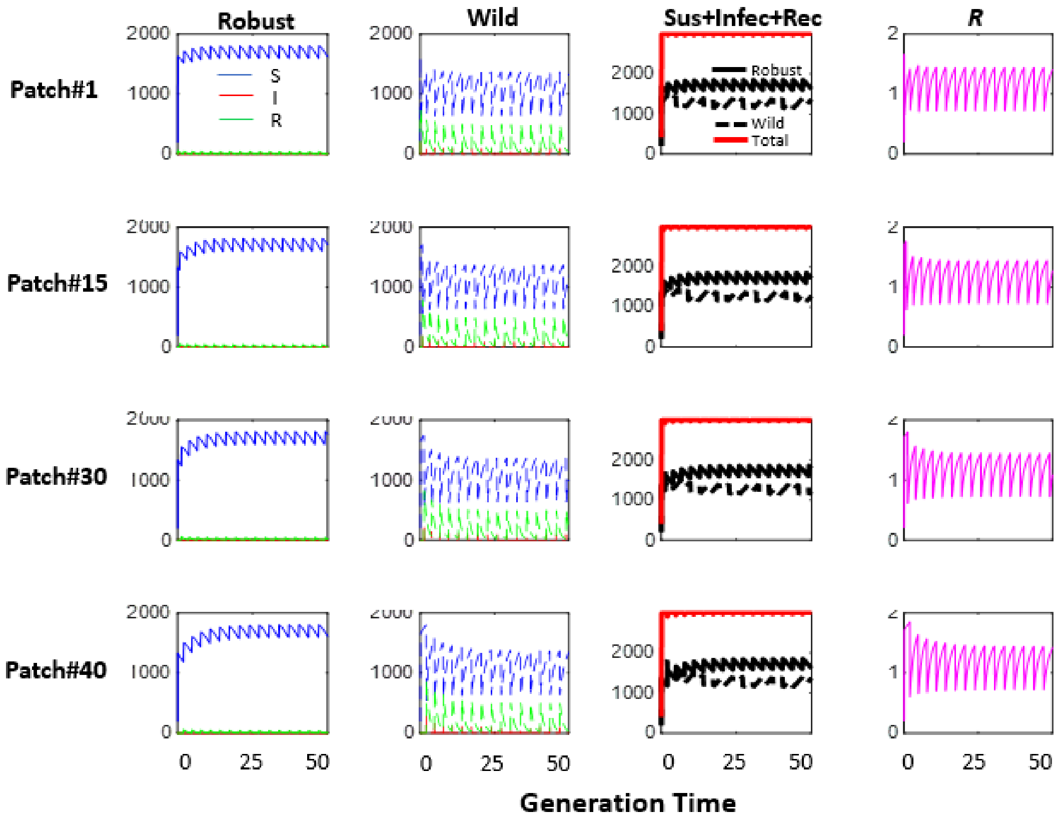

Fig. S8 The dynamics of Susceptibles, Infected, Recovered in robust type and wild type, population size in wild type, robust and total host, and effective reproductive number R in four patches labelled #1, #15, #30 and #40 when Patch B was assigned #2, #3 along the direction of disease transmission (see Fig. 6). S is blue color, I is in red, R is in green with solid in robust and dashed in wild type. All the parameters are: $r_R = 0.16$, $r_W = 0.2$, $r_{Wd} = r_{Rd} = 0.01$, $r_{Wr} = r_{Rr} = 0.2$, $\alpha_W = \alpha_R = 0.05$, $\mu_W = \mu_R = 0.0005$, $\gamma_W = \gamma_R = 0.05$, $\beta_{RR} = \beta_{WR} = 0.000005$, $\beta_{RW} = \beta_{WW} = 0.0001$ and $mig(k,j) = 0.000005$ for each pathway between focal patch #1 and B, $mig(k,j) = 0.00001$ between other patches. Initial susceptibles of both types are 200 in each patch, initial infected number of each host type is 5 in #1 patch where disease starts.

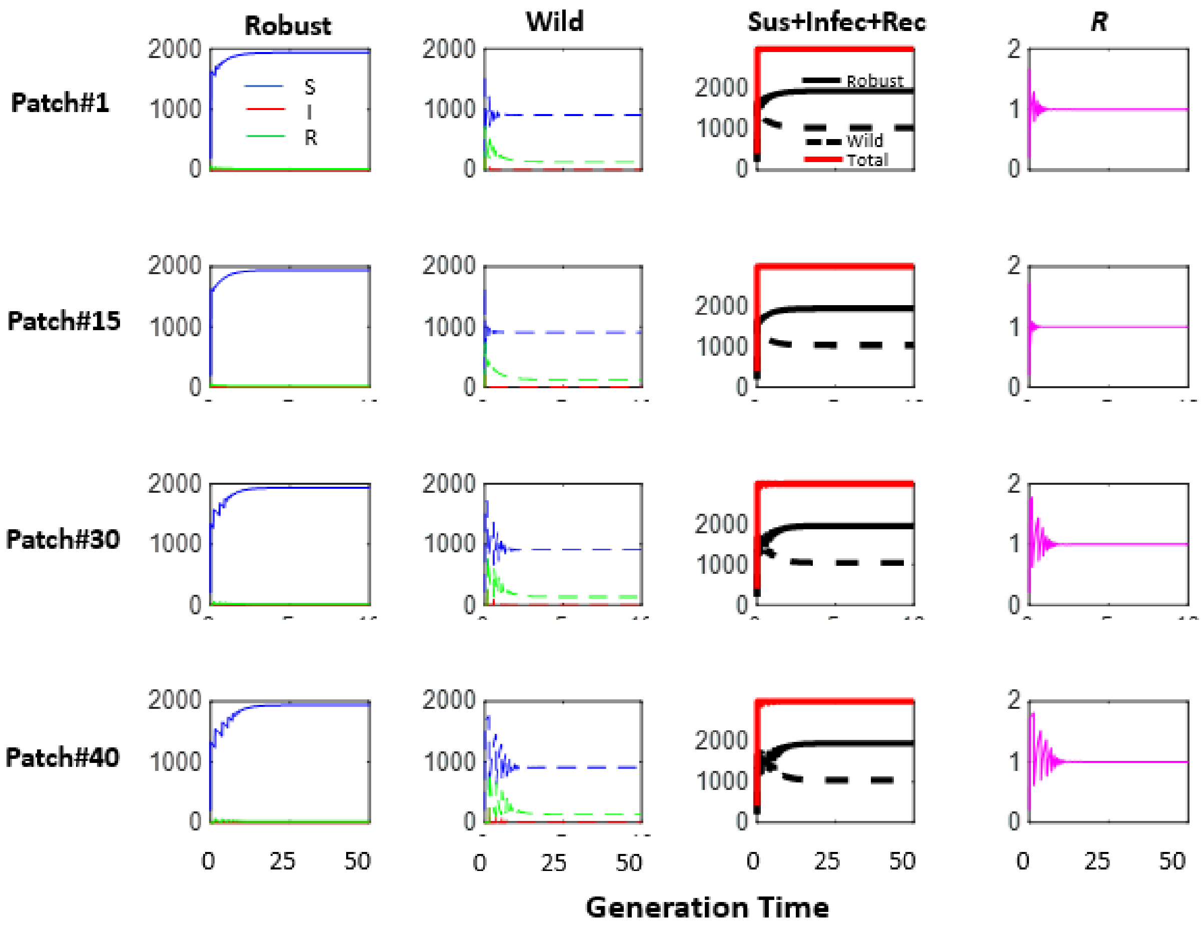

Fig. S9 The dynamics of Susceptibles, Infected, Recovered in robust type and wild type, population size in wild type, robust and total host, and effective reproductive number R in four patches labelled #1, #15, #30 and #40 when Patch B was assigned #15, #20 along the direction of disease transmission (see Fig. 6). S is blue color, I is in red, R is in green with solid in robust and dashed in wild type. All the parameters are: $r_R = 0.16$, $r_W = 0.2$, $r_{Wd} = r_{Rd} = 0.01$, $r_{Wr} = r_{Rr} = 0.2$, $\alpha_W = \alpha_R = 0.05$, $\mu_W = \mu_R = 0.0005$, $\gamma_W = \gamma_R = 0.05$, $\beta_{RR} = \beta_{WR} = 0.000005$, $\beta_{RW} = \beta_{WW} = 0.0001$ and $mig(k,j) = 0.000005$ for each pathway between focal patch #1 and B, $mig(k,j) = 0.00001$ between other patches. Initial susceptibles of both types are 200 in each patch, initial infected number of each host type is 5 in #1 patch where disease starts.

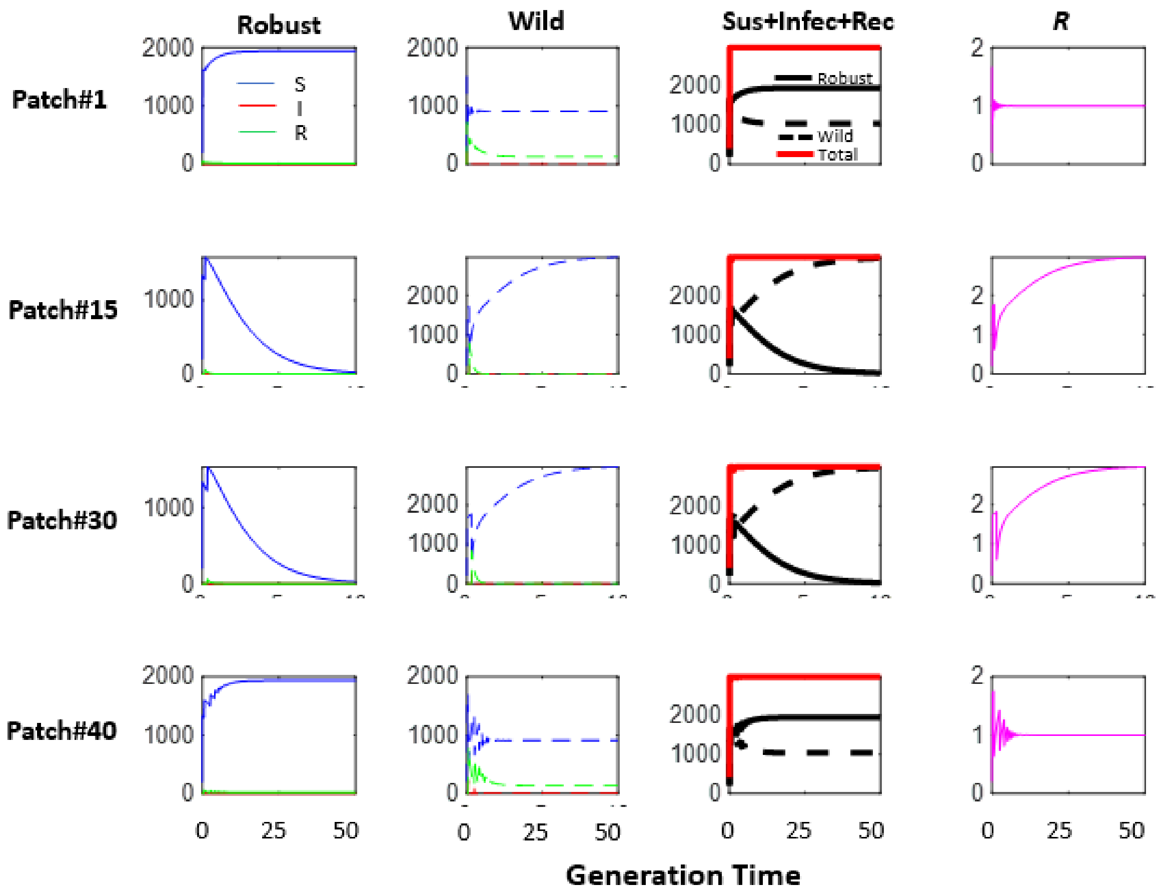

Fig. S10 The dynamics of Susceptibles, Infected, Recovered in robust type and wild type, population size in wild type, robust and total host, and effective reproductive number R in four patches labelled #1, #15, #30 and #40 when Patch B was assigned #28 along the direction of disease transmission (see Fig. 6). S is blue color, I is in red, R is in green with solid in robust and dashed in wild type. All the parameters are: $r_R = 0.16$, $r_W = 0.2$, $r_{Wd} = r_{Rd} = 0.01$, $r_{Wr} = r_{Rr} = 0.2$, $\alpha_W = \alpha_R = 0.05$, $\mu_W = \mu_R = 0.0005$, $\gamma_W = \gamma_R = 0.05$, $\beta_{RR} = \beta_{WR} = 0.000005$, $\beta_{RW} = \beta_{WW} = 0.0001$ and $mig(k,j) = 0.000005$ for each pathway between focal patch #1 and B, $mig(k,j) = 0.00001$ between other patches. Initial susceptibles of both types are 200 in each patch, initial infected number of each host type is 5 in #1 patch where disease starts.

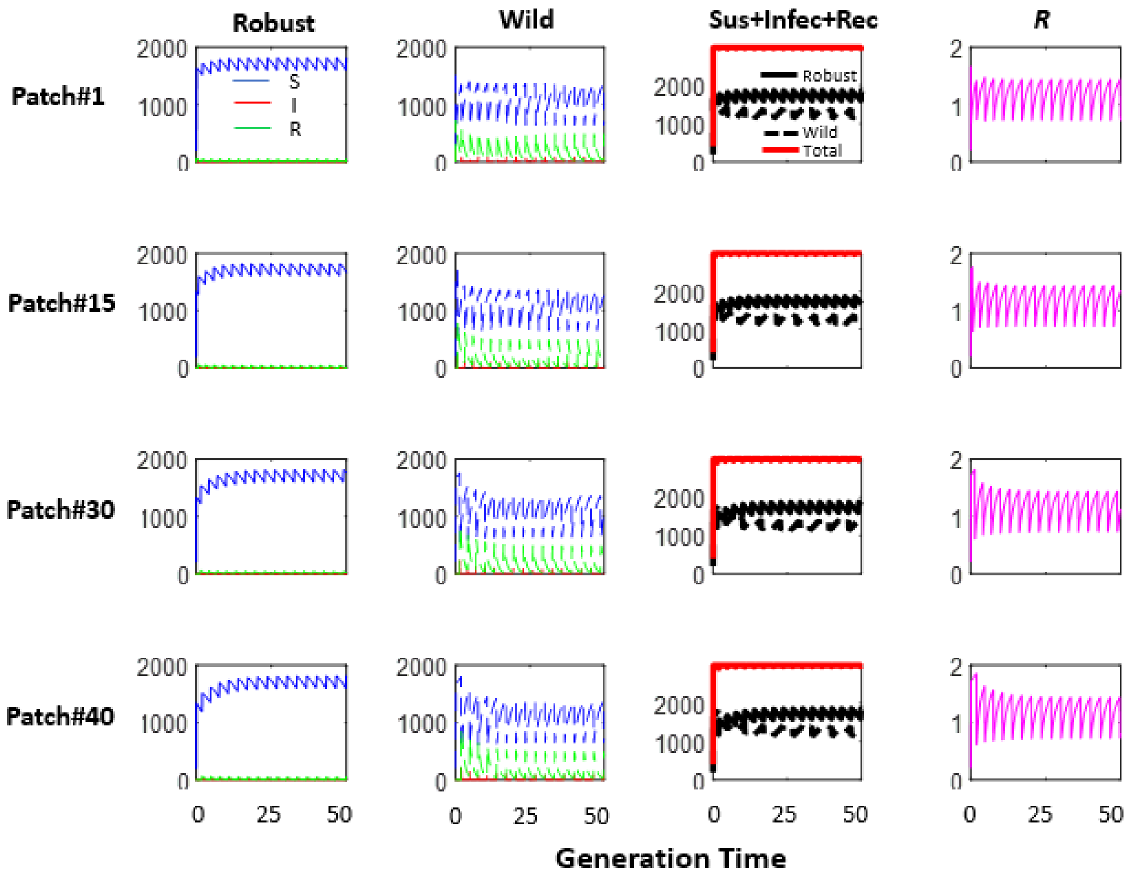

Fig. S11 The dynamics of Susceptibles, Infected, Recovered in robust type and wild type, population size in wild type, robust and total host, and effective reproductive number R in four patches labelled #1, #15, #30 and #40 when focal patch #1 connected to both patches #2 and #3 along the direction of disease transmission (see Fig. 6). S is blue color, I is in red, R is in green with solid in robust and dashed in wild type. All the parameters are: $r_R = 0.16$, $r_W = 0.2$, $r_{Wd} = r_{Rd} = 0.01$, $r_{Wr} = r_{Rr} = 0.2$, $\alpha_W = \alpha_R = 0.05$, $\mu_W = \mu_R = 0.0005$, $\gamma_W = \gamma_R = 0.05$, $\beta_{RR} = \beta_{WR} = 0.000005$, $\beta_{RW} = \beta_{WW} = 0.0001$ and $mig(k,j) = 0.000003$ for each pathway between focal patch #1 and B, $mig(k,j) = 0.00001$ between other patches. Initial susceptibles of both types are 200 in each patch, initial infected number of each host type is 5 in #1 patch where disease starts.
